# Poster Presentations - Abstracts of the 25^th^ Annual Congress of the SBRA and the 15^th^ REDLARA Taller General Rio de Janeiro/RJ - Brazil, 2021

**DOI:** 10.5935/1518-0557.20210105

**Published:** 2022

**P-01. Impact of oocyte vitrification and supplementation of the vitrification media with antioxidants and fatty acids on the lipid profile of mice blastocysts**

**Thalita S. Berteli** ^**1**^ **, Eduardo D. Borges** ^**1**^ **, Caroline M. Da Luz** ^**1**^ **, Alessandra A. Vireque** ^**2**^ **, Paula A. Navarro** ^**1**^

^*1*^ *Faculdade de Medicina de Ribeirão Preto, Universidade de São Paulo - Departamento de Ginecologia e Obstetrícia, Ribeirão Preto - SP, Brazil.*

^*2*^ *Invitra - Assisted Reproductive Technologies LTD, Supera Innovation and Technology Park, Ribeirão Preto - SP,Brazil.*

**Objective:** To investigate the impact of oocyte vitrification on lipid profile of mice blastocysts by comparing blastocysts originated from fresh and from vitrified oocytes subjected to *in vitro* fertilization. Also, to assess the effects of supplementing the vitrification media with antioxidants and unsaturated fatty acids by comparing blastocysts vitrified with Irvine Scientific, a commercial standard vitrification media; Tvitri-4, produced in small scale for research by INVITRA^®^, based on the standard composition with four modifications including carbohydrate trehalose instead sucrose, reduced non-permeant cryoprotectant concentration, and addition of two aminoacids; and Tvitri-4 supplemented with L-carnitine (LC) and oleic and linoleic fatty acids (FA).

**Methods:** Experimental study including 23 C57BL/6J mice females superovulated with 5UI eCG followed by 5UI hCG. Oocytes (n=562; 4 replicates) were randomly divided in 4 groups: fresh control group (FC), vitrified using Irvine (IRV), Tvitri-4 (T4), or supplemented Tvitri-4 (T4-LC/FA) media. Fresh or vitrified-thawed oocytes were inseminated with spermatozoa and cultivated for 96 or 120 hours in KSOM (Cosmo bio co., LTD) incubated at 37oC with 5% CO2. Blastocysts of each group were individually fixed in methanol/water. Lipids were extracted using methanol (9 blastocysts/group) and flow injected into a triple quadrupole spectrometer equipped with an electrospray ion source. Lipids were analyzed using the multiple reaction monitoring profiling (MRM-profiling) method and values of relative intensities of ions detected in each group were compared using univariate (one-way ANOVA, volcano plot) and multivariate analysis (PLS-DA).

**Results:** One-way ANOVA (*p*-value ≤0.05) showed that 90 out of the 125 lipids were differently expressed among the four groups, while a comparison between the vitrified groups showed no difference. Two by two comparisons between the control and vitrified groups using volcano plot (*p*-value ≤0.05, fold-change ≥1.5) detected 55, 17, and 11 significant features between FC vs. IRV, FC vs. T4, and FC vs. T4-LC/FA, respectively; all of them more abundant in the FC group. Partial least square discriminant analysis (PLS-DA) variables of importance (VIP scores) higher than 1.3 followed the same pattern and identified the phosphatidylinositol containing 36 carbon and one unsaturation in the fatty acyl residues - PI(36:1), and phosphatidylcholines PC(38:4), PCo(36:5), PC(34:1), and PC(30:0) among the top features; the exception being the free stearic acid (C18:0), which was the top feature in the FC x T4-LC/FA comparison, being more abundant in the latter.

**Conclusion:** Vitrification changed the lipid profile of mice blastocysts causing an overall reduction on lipid abundances that affected PC lipids the most. This effect was more apparent in IRV, followed by T4, and T4-LC/FA suggesting that supplementation of media with L-carnitine and unsaturated fatty acids may have protective effects on lipid content of blastocysts from vitrified oocytes, whose impacts needs further investigation.

**Supported by:** Coordination for the Improvement of Higher Education Personnel (CAPES, Finance Code: 001, process number 88887.371487/2019-00, and process n. 88887.597054/2021-00); Foundation to Support Teaching, Research, and Assistance (FAEPA) at Clinical Hospital, Faculty of Medicine of Ribeirão Preto, University of São Paulo; Invitra Assisted Reproductive Technologies LTDA; and Brazilian National Council for Scientific and Technological Development (CNPq), (process n. 305173/2019-7).

**P-02. Online PEC for nurses in assisted reproduction, an experience report**

**Fernanda Kunrath Robin** ^**1**^ **, Mariane Féix da Cunha** ^**1**^ **, Joana Gabrielle Carvalho Brandolt Chagas** ^**1**^ **, Nilo Frantz** ^**1**^ **, Gabriella Mamede Andrade, Luiza Mezzomo Donatti** ^**1**^

^*1*^ *Nilo Frantz Medicina Reprodutiva. Porto Alegre, RS, Brazil.*

**Objective:** The development of the field of work for nurses in assisted human reproduction has exacerbated the need to develop research, specialization and continuing education in the area. Thus, we thought about describing the experience of two nurses who were part of the first group of the Continue Education Program of the Red Latinoamericana de Reproducción Asistida, entitled PEC ONLINE for Nurses, Matrons, Midwives and Scrub nurse. The objective of this work is to demonstrate the real need for continue education programs as well as specializations in the field of nursing.

**Methods:** Based on PEC ONLINE, we thought about describing the experience from the point of view of two nurses. Context description: Assisted human reproduction is a highly specialized area that, in most cases, is not covered in undergraduate nursing disciplines. The arrival of professional nurses to assisted human reproduction took place slowly and gradually, as there was no knowledge to effectively perform nursing care for couples who sought human reproduction clinics. For this reason, two nurses, from the same group, but working in different states, Rio Grande do Sul and São Paulo, participated in the PEC ONLINE for nurses, matrons, midwives and surgical scrub nurses. Nurses have an average of nine years of experience in direct care of infertile patients. The course lasted approximately 12 months, being divided into seven modules. Each module was the responsibility of a teacher, being teachers from different countries of latin american. Each of the modules developed during the continuing education program contemplated a different subject of assisted human reproduction. Both nurses have specializations in other related areas, but it was the first experience in a program aimed at this specific area.

**Results:** During all the time, these nurses worked, much of the knowledge acquired was through research in databases and much through daily work routines. Precisely for not having a pre-established systematization of nursing care, each clinic works according to its daily reality, developing care in the best way for its patient. The opportunity to participate in this continuing education program brought, in addition to technical knowledge, exchanges of valuable experiences for the care work. It was a moment in which we stopped to reflect on the way we act and how we can evolve in the treatment of these patients who, in most cases, are very suffering to us. In addition, the course enabled interaction with nurses, matrons, midwives and surgical scrub nurses who are very experienced in the field of assisted reproduction nursing, and they were able to show the different ways in which these professionals work in Latin America. All the knowledge acquired helped to bring more tranquility and security in working with patients.

**Conclusion:** Each profession in the health area has its way of describing what it knows and how it acts in relation to its knowledge. Nurses deal with responses to health problems/life processes among individuals, families, groups and communities, and these responses are the central concern of nursing care. In human reproduction it is no different, a multidisciplinary team works towards the realization of the dream of building a family. In this way, this course allowed a great gain, not only for the professional nurse, but also for the multidisciplinary team and patients. This experience report shows the importance of courses on assisted human reproduction aimed at nurses and the need to think of other ways that can bring information and systematization of care to these professionals.

**P-03. Endometriosis in patients undergoing treatment of Assisted Human Reproduction**

**Andrea Mesquita Lima** ^**1**^ **, Gleicyane Sousa dos Santos** ^**1**^ **, Sebastião Evangelista Torquato Filho** ^**1**^ **, Fábio Eugênio Magalhães Rodrigues** ^**1**^ **, Marcus Aurélio Bessa Paiva** ^**1**^ **, Tulius Augustus Ferreira de Freitas** ^**1**^ **, Eduardo Gomes Sá** ^**1**^

^*1*^ *BIOS - Centro de Medicina Reprodutiva do Ceará.*

**Objective:** Endometriosis is an inflammatory disease characterized by the presence of the endometrium outside the uterine cavity, making it thicker and more amenable to adherence to other abdominal organs. The tubal factor is the main factor of infertility associated with endometriosis, because due to the inflammatory process caused by the disease, anatomical alteration of the tubes may occur, which can hinder natural fertilization. The objective of this work was to analyze the extent to which endometriosis correlates with the results of in vitro fertilization treatment, comparing the rates of oocyte recovery, maturity rate, fertilization rate, blastulation rate and euploidy rate of patients with and without endometriosis.

**Methods**: Retrospective study, conducted from January 2021 to July 2021, in which cases of 102 patients undergoing assisted reproduction treatment were analyzed. The patients were divided into two groups: Group of patients without endometriosis (n=55) and Group of patients with endometriosis (n=47). Patients from 40 years of age and also those whose partner presented male factor were excluded from the analysis.

**Results:** The egg recovery rate was 96% in the group without endometriosis and 95% in the group with endometriosis. The maturity rate was 78% in the group without endometriosis, and 75% in the group of patients with endometriosis. The fertilization rate was 88% in the group without endometriosis, and 85% in the group with endometriosis. The blastulation rate was 61% in the group without endometriosis, and 51% in the group with endometriosis. The euploidy rate was 47% in the group without endometriosis, and 45% in the endometriosis group.

**Conclusion:** Oocyte recovery rates, maturity rate, fertilization rate and euploidy rate did not show significant difference between the groups analyzed. However, a lower rate of blastocyst formation was found in the group of patients affected with endometriosis. The reduced blastocyst formation rate in this group of patients can be attributed to a possible oxidative stress that the egg may suffer in the follicular environment, which can impair nuclear maturation, and interfere with blastocyst formation. In view of these findings, it can be concluded that the cumulative pregnancy rate in the group of patients affected with endometriosis may be lower, since they have a smaller number of embryos available.

**P-04. The influence of the oocyte with large perivitelline space in fertilization, cleavage and blastocyst formation rate in different groups of age**

**Camila Dutra de Souza Francisquini** ^**1**^ **, Vinicius Bonato da Rosa** ^**1**^ **, Alessandro Gomes Schuffner** ^**1**^

^*1*^ *Clinica Conceber - Centro de Medicina Reprodutiva.*

**Objective:** The objective of this study was to evaluate fertilization, cleavage, and blastocyst formation rates arising from oocytes with large perivitelline space (large PVS) in different age groups. Furthermore, if these rates differ when compared to the morphologically normal oocyte.

**Methods:** This retrospective study compiled the results of oocyte retrieval and embryo development couples subjected to fertilization in vitro from 2014 to 2020. Our study group was 24 to 47 years old, under IVF fresh cycle, with fresh semen. The Oocyte recovery procedure resulted in a total of 3825 mature oocytes. The selected oocytes were divided into 2 initial groups, classified as morphologically normal (n=2294) and oocytes with large PVS (n=452). They were disporting according to the women's age: <35 years; 35 - 37 years; 38 - 40 years; 41-42 years and >42 years, following the Society for Assisted Reproductive Technology (SART) criteria. ICSI was performed and the fertilization, cleavage, and blastocyst formation rates were calculated between the age groups and within each age group for normal and large PVS oocytes. Rates (%) were compared between groups using the Z-test statistical method for 2 proportions, adopting a statistically significant *p*<0.05.

**Results:** Of all mature oocytes recovered the morphologically normal represented 60% of the total, in addition, the most frequent defect, performing 12% of total recovery oocytes was large PVS. In the comparison by age group, the fertilization rate of oocytes with large PVS was significantly lower (*p*<0.05) in the >42 years group (36%). However, in the <35 years, 35-37 years, 38-40 years and 41-42 years groups the fertilization rate was 68%, 68% 72% and 73%, respectively. The cleavage rate fluctuated by 88 to 100% among the age groups and didn't show a statistical difference (*p*>0.05). Similar data were observed comparing the blastocyst formation rate (<35 years group, 54%; 35-37 years group, 50%; 38-40 years group, 55%; 41-42 years group, 58% and >42 years group, 50%), showing no statistical difference between them (*p*>0.05). However, when we compared the fertilization rate between oocytes with large PVS versus normal oocyte of >42 years women's group, it was observed that the fertilization rate of oocytes with large PVS (36%) was statistically lower (*p*<0.05) when compared to normal oocytes (73%), this parameter also showed a statistical difference in the <35 years women group (68% and 94%, respectively). The blastocyst formation rate in the >42 years group was statistically lower (*p*<0.05) in the large PVS oocytes (50%) when compared to normal oocytes (59%). For the other age groups, no statistical differences were observed in fertilization, cleavage, and blastocyst formation rate in comparisons between large PVS or normal oocytes.

**Conclusion:** Based on the presented results it's possible to conclude that the defect most commonly observed among the oocytes analyzed was the large PVS. Moreover, the large PVS oocytes showed a negative influence on fertilization rate in the <35 and >42 years group. On the other hand, when we use the blastocyst formation rate, the negative influence is only observed in the group with >42 years old. Therefore, oocytes with large PVS predict a poor prognosis for embryonic development in these age group patients.

**P-05. Comparison of single versus dual trigger protocols in assisted reproduction treatments**

**Vinicius Bonato da Rosa** ^**1**^ **, Camila Dutra de Souza Francisquini** ^**1**^ **, Alessandro Gomes Schuffner** ^**1**^

^*1*^ *Clinica Conceber - Centro de Medicina Reprodutiva.*

**Objective:** Currently, the profile of patients attempting assisted reproduction treatment has changed, younger women are seeking to preserve fertility by freezing eggs, while women attempting pregnancy are getting older. With this in mind and seeking the best treatment profile for our patients, we seek to identify the best protocol to optimize treatment without increasing the risks for our patients. The trigger protocol is a decisive step for the success of the procedure when we think about oocyte recovery and maturity. The present study aimed to compare the number of retrieved oocytes and the number of mature oocytes in patients who received a single trigger versus patients who received a dual trigger.

**Methods:** This retrospective research compiled data from patients aged 22 to 47 years who underwent assisted reproduction treatment from January 2019 to August 2021. The patients were divided into 2 groups, group 1 (n=199 patients) who a single trigger was administered; and group 2 (n=242 patients) in which patients received dual trigger. The follicular aspiration procedure was performed 35 hours after the trigger administration. First of all, the mean age of the patients and the number of follicles with 14mm or more on the day of the trigger were calculated to confirm the homogeneity of the groups. In addition, the number of oocytes retrieved after aspiration and the number of mature oocytes was compared. The means of each variable were compared with the One-way ANOVA test for independent samples, adopting the statistical *p*<0.05.

**Results:** When we evaluated the mean age (± standard deviation) of patients in group 1 who received a single trigger, 37.52 (±4.47) years and in group 2 who received dual trigger, 37.79 (±4.37) years, we did not observe the statistical difference (*p*>0.05) between them. A similar result was reached when comparing the number of follicles, which for group 1 was 1741, with a mean (± standard deviation) of 8.78 (±6 .69) and in group 2, a total of 2306 follicles with an average of 9.52 (±7.05), which confirmed the homogeneity between the groups. For the variable oocytes recovered after aspiration, group 1 had 7.63±6.39 oocytes while group 2 had 8.50±6.70 oocytes, with no statistical difference (*p*>0.05). The identical result was obtained when comparing groups 1 and 2 concerning mature oocytes, with means of 5.78±5.20 and 6.50±5.49, respectively. However, there was no significant difference between the groups.

**Conclusion:** From the results obtained we can conclude that although a favorable trend is visible when evaluating the recovery of eggs and mature eggs in the group that used double trigger, however, the results were not statistically significant.

**P-06. How men who are referred for IVF cope with infertility stress**

**Cássia Cançado Avelar** ^**1**^ **, Ricardo Mello Marinho** ^**1**^ **, Leonardo Matheus** ^**1**^ **, João Pedro Junqueira Caetano** ^**1**^

^*1*^ *Clínica Huntington/Pró-Criar, Belo Horizonte/MG.*

**Objetive:** To evaluate the perception of stress and coping strategies used by men during an IVF treatment.

**Methods:** Cross-sectional study with 120 men, in a private reproductive medicine clinic in Belo Horizonte/Brazil. This research was approved by Plataforma Brasil - CONEP (CAAE 46282614.0.0000.5134).

Participants completed the Demographic/Clinical Questionnaire: gender (category that refers to biological sexual condition, categorized in male or female); age (in full years, at the time of the interview, referred by the subject); education (categorized in incomplete primary education and complete, incomplete and complete secondary education, higher and postgraduate education); with or without children (reference to having children, categorized as biological child and adopted child); duration of infertility (time the person said they were trying to get pregnant current partner); infertility factor (variable obtained from the question about the reason for the difficulty in getting pregnant or getting the partner pregnant, categorized in infertility factors) and previous assisted reproduction treatments (reference to having performed previous pregnancies, specifying what treatment). 

The Fertility Problems Inventory, a 6-point Likert scale, produces a global infertility stress score (total score measuring overall infertility-related stress) in addition to five sub scores on scales measuring: social concern (sensitivity to comments, reminders of infertility, feelings of social isolation, alienation from family or peers), sexual concern (diminished sexual enjoyment or sexual self-esteem, scheduled sexual relations difficult), relationship concern (difficulty talking about infertility, understanding/accepting sex differences, concerns about impact on relationship), need for parenthood (close identification with role of parent, parenthood perceived as primary or essential goal in life) and rejection of childfree lifestyle (negative view of childfree lifestyle or status quo, future satisfaction or happiness dependent on having a child (or another child)). 

The Coping Strategies Inventory, a 4-item Likert scale ranging and includes eight subscales: confrontative coping (directly challenging the stressor); distancing (making light of the infertility); self-controlling (keeping feeling about the infertility to oneself and trying to keep these feelings from interfering with daily activities); seeking social support (talking to friends or professionals about the infertility); accepting responsibility (believing one is responsible for the infertility); escape/avoidance (avoiding people and reminders of the infertility); problem-solving (taking action towards finding a solution to the infertility) and positive reappraisal (reevaluating the experience of infertility to find unexpected benefits or personal growth). 

Data were collected between October 2015 and February 2017

**Results:** The mean age was 37.34 years, and most individuals had higher education (49.58%) and postgraduate education (23.75%). Regarding children, 83.75% did not have them. The infertility factors reported were 37.92% female, 26.67% with no apparent cause, 25% male and 10.42% mixed. Regarding previous treatments, the majority (56.25%) had already undergone some assisted reproduction treatment. In the correlation of global stress with demographic and clinical variables, only in the age variable, there was a significant (*p* = 0.038) and negative (r = -0.13) correlation; therefore, the older the individual, the lower the overall infertility stress tends to be. The infertility problems more stressful were rejection of childfree lifestyle (4.67) and the need for parenthood (3.27), while social infertility stress were the ones that generated less stress (2.17).Regarding coping strategies, the most used were problem- solving (1.93) and self-controlling (1.35) and the least used were confrontative coping (0.40), distancing (0.72) and accepting responsibility (0.72). The coping strategies more stressful were seeking social support, escape/avoidance, problem-solving and positive reappraisal, with these correlations being significant (*p* value <0.001) and positive (r>0.00); thus, the greater any one of these indicators, the greater the stress and vice versa.

**Conclusion:** the study highlights how men referred for IVF cope with infertility, and how coping is related to infertility stress. Results of the study can be considered by practitioners and clinicians who work with men undergoing IVF.

**P-07. OsteraTest - A novel non-invasive tool for oocyte selection using gene expression and artificial intelligence**

**Lucia von Mengden** ^**1,2,3,4**^ **, Marco Antônio De Bastiani** ^**1,2,3,4**^ **, Carlos Alberto Link** ^**4,5**^ **, Leticia Schmidt Arruda** ^**5**^ **, Fábio Klamt** ^**1,2,3,4**^

^*1*^ *Laboratory of Cellular Biochemistry, Department of Biochemistry, ICBS/UFRGS, Porto Alegre (RS), Brazil.*

^*2*^ *National Institutes of Science & Technology - Translational Medicine (INCT- TM), Porto Alegre (RS), Brazil.*

^*3*^ *Postgraduate Program: Biochemistry, Universidade Federal do Rio Grande do Sul (UFRGS), Porto Alegre (RS), Brazil.*

^*4*^ *Ostera, Porto Alegre (RS), Brazil.*

^*5*^ *Clínica ProSer, Porto Alegre (RS), Brazil.*

**Objective:** Proper oocyte selection is an important bottleneck for In Vitro Fertilization (IVF) success. Nowadays, oocyte selection relies mainly in morphological analyses, which is not an unbiased method and may fail to reveal the real competence status of gametes. In addition, the trend towards laws restricting the number of eggs to be fertilized and the increasing demand for egg freezing create a demand for techniques to accurately predict the developmental potential of embryos before fertilization. Cumulus oophorus cells (CC) are somatic cells that surround the oocyte at the antral follicle. Attached to the oocyte through specialized junctions, the CCs provide essential compounds to the oocyte, receives signals from the gamete and provides protection. It is directly involved in oocyte maturation and development, and thus is a valuable non-invasive source of biological information regarding the oocyte’s health. Artificial intelligence can be used to identify key biological processes and markers of interest through machine learning methods. This strategy has been applied successfully in distinct fields of biomedical sciences, such as neuroscience and oncology. In this context, we performed differential expression analysis of human CC datasets and machine learning to identify genes and expression patterns that could predict oocyte quality, developing a tool that could be easily applied in the clinical environment. Afterwards, the prediction models obtained were validated in house with a cohort patient samples.

**Methods:** This study was approved by the Research Ethics Committee (#68081017.2.0000.5347). Gene Expression Omnibus (GEO) publicly available microarray data (GSE27377) composed of 80 CC samples related to oocytes yielding top quality blastocysts and CC related to oocytes that did not reach blastocyst stage was used to compute the models of oocyte quality prediction using machine learning methods. Thirty-six isolated CC samples from each oocyte of 18 patients submitted to Intracytoplasmic Sperm Injection (ICSI) procedure were used for the validation phase of this study. Samples were divided in two groups: 18 CCs from oocytes that developed into top quality blastocysts in day 5 after ICSI and 18 CCs from oocytes that presented arrested development. Samples were submitted to real time quantitative PCR. Afterwards, gene expression levels for each gene and sample were submitted to the final software product, the OsteraTest, in a double-blind approach. The software indicated the development potential of each oocyte and this ranking was compared to the embryologist’s day 5 blastocyst classification according to Gardner.

**Results:** The bioinformatic approach implemented resulted in the OsteraTest, composed of 8 machine learning models using a 25-gene network that altogether can predict oocyte quality, thus representing a very complex assembly. The software presented more than 72% efficacy in predicting the oocyte developmental capacity into a top-quality day 5 blastocyst.

**Conclusion:** Oocyte quality prediction and embryo selection for transfer are of great interest for patients and assisted reproductive clinics that aim for better success rates in IVF treatments. The OsteraTest proved to be a valuable non-invasive tool to predict embryo formation and oocyte capacity even before fertilization. It can enable the clinics to anticipate successful treatments and provide a predictive report for oocyte freezing patients. Our group is currently developing a large scale prospective randomized study for further validation of findings.

**P-08. Congenital malformations in IVF/ICSI patients in a Brazilian cohort**

**Edilberto Araújo Filho** ^**1**^ **, Leonardo Previato de Araújo** ^**1**^ **, Cássio Leão Fácio** ^**1**^ **, Ligiane Alves Machado-Paula** ^**1**^ **, Ligia Fernanda Previato de Araújo** ^**1**^

^*1*^ *Centro de Reprodução Humana de São José do Rio Preto (CRH Rio Preto), São José do Rio Preto, SP, Brasil.*

**Objective:** The objective of this study is to evaluate the incidence of congenital malformations (CM) in a cohort of Brazilian patients since some publications (Qin et al., 2016) have shown that the incidence of CM varies from country to country and very little publications address Brazilian population in this matter.

**Methods:** This is a cohort study with a population of IVF/ICSI couples who had their babies in the period of January/2010 to December/2019. A total of 560 couples were interviewed online (by zoom) by a single geneticist examiner in the presence of the children borned. As a control group we used the ECLAMC - 'Estudio Colaborativo Latino Americano de Malformaciones Congenitas', the Latin-American collaborative study of congenital malformations.

**Results:** A total of 15 children (2.5%) had major CM: 5 cases of Down syndrome; 3 cases of urogenital malformation (unilateral renal agenesis with bladder agenesis, double ureter and ureterocele); 1 case of gastrointestinal malformation (esophageal hernia); 1 case of Edwards syndrome; 1 case of microcephaly due to encephalocele; 1 case of Turner syndrome; 1 case of congenital clubfoot; 1 case of cleft palate and 1 case of Townes-Brocks syndrome. The incidence of major CM in the ECLAMC is 2.1% (*p*>0.05). In the 15 children born with CM (major), 3 came from twin gestation and one from a triplet gestation. In the 9 cases of abortion with abnormal karyotype, 5 the mothers were ≥ 38 years of age. We had 2 cases of babies born with minor CM: 1 case of cheek asymmetry and one case of sectoral heterochromia. We had 15 cases of abortion in the first trimester that underwent genetic tests (karyotype) which represents 6.5% of all abortions tested: 6 had normal karyotype; 3 had Down syndrome; 2 Turner syndrome; 1 Trisomy 16; 1 Trisomy 2; 2 Trisomy 8, for an incidence of abnormal karyotype of 60% in the abortion group.

**Conclusion:** The incidence of CM in IVF/ICSI babies born in a Brazilian cohort population is not statistically different when compared with babies born by natural conception showing that each country should evaluate its own population to better inform patients undergoing IVF treatment.

**P-09. Association between the duration of equilibration step of the vitrification and changes of oocyte membrane lipids**

**Thalita S. Berteli** ^**1**^ **, Eduardo D. Borges** ^**1**^ **, Caroline M. Da Luz** ^**1**^ **, Paula A. Navarro** ^**1**^ **, Alessandra A. Vireque** ^**2**^

^*1*^ *Faculdade de Medicina de Ribeirão Preto, Universidade de São Paulo - Departamento de Ginecologia e Obstetrícia, Ribeirão Preto - SP, Brazil.*

^*2*^ *Invitra - Assisted Reproductive Technologies LTD, Supera Innovation and Technology Park, Ribeirão Preto - SP, Brazil.*

**Objective:** Volumetric damages and cytotoxicity due to cryoprotectants exposure are time-sensitive in the vitrification process and can trigger the lipid remodeling of the plasma membrane. Here, we investigate the effects of exposure time to equilibration solution (ES) on membrane lipid profile of C57BL/6J mice oocytes using MRM-profiling, a sensitive exploratory method for lipidomics of oocytes and embryos1. We also assessed the effects of supplementing ES with antioxidants and unsaturated fatty acids. Experimental groups included oocytes equilibrated with Irvine Scientific (IRV), a commercial standard vitrification medium; Tvitri-4 (T4) produced in small scale for research by INVITRA^®^, and Tvitri-4 supplemented with Lcarnitine (LC) and oleic and linoleic fatty acids (FA) (T4-LC/FA; Patent n.BR102019013697-9).

**Methods:** Experimental study including oocytes randomly divided in 7 groups: non-exposed (NE) and exposed to either 10-minute equilibration step (ES10) according to the manufacturer’s procedure or 7-minute equilibration step (ES7), using IRV, T4, or T4-LC/FA media. After equilibration, oocytes were washed in methanol:H2O, and lipids were extracted with methanol. Diluted lipid extracts were flow injected into the triple quadrupole spectrometer with electrospray ionization (ESI) source. Relative ion intensities were used for univariate (ANOVA, fold-change, t-test, volcano plot) and multivariate analysis (PCA, PLS-DA, cluster analysis). Most informative lipids were sorted out using statistical significance of p<0.05 or partial least square discriminant analysis (PLS-DA) variables of importance (VIP) scores>1.

**Results:** One-way ANOVA showed PC(38:8) differently represented among NE, ES7 and ES10 groups, upregulated in T4-LC/FA oocytes. In ES7 groups, two by two comparisons between IRV, T4 and T4-LC/FA using volcano plot (p-value≤0.05; foldchange≥2.0) 4, 5, and 4 significant lipids were detected between IRV vs. T4, IRV vs. T4-LC/FA, and T4 vs. T4-LC/FA, respectively, with increased abundance of PEo(36:1) overrepresented in IRV (fold-change=12.5). In ES10 groups, 11, 19 and 4 lipids were differentially represented among IRV vs. T4, IRV vs. T4-LC/FA, and T4 vs. T4-LC/FA, respectively. PLS-DA VIP scores identified in IRV oocytes 8 free fatty acids, phosphatidyletanolamine PEo(36:1), phosphatidylcholines PCo(32:0) and PC(36:3), phosphatidylserines PS(14:1), PS(16:0), PS(30:2), PSo(40:5), and phosphatidylinositols PI(22:4) and PI(40:8) among the top features, whereas unsaturated PC and SM predominated in T4 and T4-LC/FA oocytes. Significant lipids observed between T4 and T4-LC/FA pointed oleic (18:1) and linoleic (18:2) acids upregulated in T4-LC/FA oocytes exposed for 10 min to ES.

**Conclusion:** The duration of the equilibration phase changed the abundance and composition of some membrane lipids. Phospholipids were upregulated at 10-minute exposure to ES, except in IRV oocytes. The oleic and linoleic acids used as supplements in T4-LC/FA medium were internalized and metabolited by the oocytes and seem to contribute to better preserving membrane phospholipids during cell equilibration.

**Supported by:** Coordination for the Improvement of Higher Education Personnel (CAPES, Finance Code: 001, process number 88887.371487/2019-00, and process n. 88887.597054/2021-00); Foundation to Support Teaching, Research, and Assistance (FAEPA) at Clinical Hospital, Faculty of Medicine of Ribeirão Preto, University of São Paulo; Invitra Assisted Reproductive Technologies LTDA; and Brazilian National Council for Scientific and Technological Development (CNPq), (process n. 305173/2019-7).

**P-10. Comparison of Diff-quick and Spermac staining methods for sperm morphology evaluation**

**Lincoln Bastos Farias Junior** ^**1,2**^ **, Paula Fontoura Coelho de Souza** ^**1**^ **, Mariana Duque de Mello** ^**1**^ **, André Rodrigues da Cunha Barreto-Vianna** ^**3**^ **, Cristiane da Fonte Ramos** ^**4**^

^*1*^ *Rio de Janeiro Sperm Bank (Banco de Sêmen do Rio de Janeiro), Rio de Janeiro, Brazil.*

^*2*^ *Federal University of Rio de Janeiro, Rio de Janeiro, Brazil.*

^*3*^ *Department of Biosciences, Federal University of Paraná, Palotina, Paraná, Brazil.*

^*4*^ *University of Rio De Janeiro, Rio de Janeiro, Brazil.*

**Objective:** This study aimed to compare two staining methods: Diff-quick and Spermac^®^, for evaluation of human sperm morphology. Furthermore, it was intended to investigate if there is a difference in the patient´s final diagnosis in each technique.

**Methods:** Samples from 50 patients were used for this study. Two slides were made for each patient. One was stained with Diff-quick and the other with Spermac^®^. In each slide, a hundred spermatozoa were counted and classified using a optical microscope under X1,000 magnification with oil immersion.

**Results:** The two staining methods successfully highlighted the head and tail, however, Spermac^®^ stain showed a better visualization of the midpiece. There were no differences between sperm classification in the Diff-quick and Spermac^®^ staining methods when analyzing the categories: head abnormalities (93.4±0.6 % *vs*. 94.2±0.6 %; *p*=0.3665) and tail abnormalities (16.6±1.3 % *vs.* 14.8±1.3 %; *p*=0.3032). However, there was a higher percentage of spermatozoa classified as normal in Diff-quick (3.98±0.4 % *vs.* 2.8±0.3 %; *p*=0.0385); and a higher percentage of spermatozoa classified as midpiece abnormalities in the Spermac^®^ (24.8±2.0 % *vs.* 55.7±2.0 %; *p*<0.0001).

**Conclusion:** Diff-quick stain results in a higher number of spermatozoa classified as normal and this difference might be related to the midpiece abnormalities identification, which was better evident in Spermac^®^ stain. Despite having the same aim and practicality, the Spermac^®^ stain presented a better definition of the structures. The lack of definition of the midpiece stained by Diff-quick increased the incidence of spermatozoa with normal morphology, not consistent with reality.

**P-11. Effect of ovarian stimulation and in vitro fertilization on the lipid profile of mice blastocysts**

**Thalita S. Berteli** ^**1**^ **, Eduardo D. Borges** ^**1**^ **,Caroline M. Da Luz** ^**1**^ **, Alessandra A. Vireque** ^**2**^ **, Paula A. Navarro** ^**1**^

^*1*^ *Faculdade de Medicina de Ribeirão Preto, Universidade de São Paulo - Departamento de Ginecologia e Obstetrícia, Ribeirão Preto - SP, Brazil.*

^*2*^ *Invitra - Assisted Reproductive Technologies LTD, Supera Innovation and Technology Park, Ribeirão Preto - SP, Brazil.*

**Objective:** To evaluate the impact of ovarian stimulation (OS) and OS plus in vitro fertilization (IVF) procedure on lipid profile of mice blastocysts by comparing the lipid profile of blastocysts originated from natural mating with or without previous superovulation and blastocysts from oocytes of superovulated females subject to IVF.

**Methods:** Experimental study including blastocysts recovered from C57BL/6J females (8 weeks) that were mated with males after confirmation of the estrous cycle (natural in vivo control group - N) or after superovulation with 5 UI equine chorionic gonadotropin followed by 5UI human chorionic gonadotropin (superovulation in vivo control group - S). For the IVF group (IVF), oocytes collected from superovulated females were inseminated with spermatozoa and cultivated for 96 hours in continuous culture media (KSOM, Cosmo bio co., LTD) incubated at 37oC with 5% CO_2_. Blastocysts of each group were individually fixed in methanol/water. Lipids of 9 blastocysts per group were extracted using methanol. Individual samples containing the lipid extracts of blastocysts were then diluted and flow injected into a triple quadrupole spectrometer equipped with an electrospray ion source. Lipids were analyzed using multiple reaction monitoring profiling (MRM-profiling) method and values of relative intensities of ions detected in each group were compared using univariate (one-way ANOVA, volcano plot) and multivariate analysis (PCA, PLSDA, hierarchical cluster analysis).

**Results:** One-way ANOVA (*p*-value ≤0.05) showed that 95 out of the 125 lipids were differently expressed among the three groups. The top five important features identified by partial least square discriminant analysis (PLS-DA) variables of importance (VIP) scores (>1.32) were free fatty acid C28:0, sphingomyelin (SM d18:1/14:0), phosphatidylserine PS(20:4), phosphatidylcholine PCo(36:5), and phosphatidylethanolamine PE(14:1), all of which were more abundant in the IVF group, followed by S and N, respectively. This pattern was observed in all top 50 features selected by PLS-DA VIP (>1). PCA and heatmaps generated by hierarchical cluster analysis suggested that IVF was further separated from the other groups. This was confirmed by two by two-group analysis using volcano plot (*p*-value ≤0.05, fold change ≥1.5) that detected 15, 57, and 99 significant features between N *vs*. S, IVF *vs*. S, and IVF *vs*. N, respectively.

**Conclusion:** While ovarian stimulation alone promotes some alterations in lipid profile of in vivo originated blastocysts when compared with in vivo blastocysts from natural cycles, mainly on phosphatidylcholines, IVF process using oocytes from superovulated mice was responsible for more extensive changes observed on the lipid profile of the studied blastocysts, related to overall increased abundances in the relative concentration of specific membrane phospholipids and fatty acids.

**Supported by:** Coordination for the Improvement of Higher Education Personnel (CAPES, Finance Code: 001, process number 88887.371487/2019-00, and process n. 88887.597054/2021-00); Foundation to Support Teaching, Research, and Assistance (FAEPA) at Clinical Hospital, Faculty of Medicine of Ribeirão Preto, University of São Paulo; Invitra Assisted Reproductive Technologies LTDA; and Brazilian National.

**P-12. Using time lapse technology to evaluate cellular division and blastocyst formation timing in patients that obtained pregnancy**

**Shana Wander Flach** ^**1**^ **, Ricardo Marques de Azambuja** ^**1**^ **, Marta Ribeiro Hentschke** ^**1**^ **, Alvaro Petracco** ^**1**^ **, Mariangela Badalotti** ^**1**^

^*1*^ *FERTILITAT - Centro de Medicina Reprodutiva.*

**Objective:** To evaluate the cellular division timing and the time of first blastocyst formation in patients that obtained pregnancy.

**Methods:** A retrospective case-control study was performed including 172 transferred cycles, using time lapse technology. In this study, both fresh and frozen embryo transfer cycles were included, while preimplantation genetic testing (PGT), frozen oocytes cycles and egg recipients’ cycles were excluded. The samples were divided into two groups: patients that obtained pregnancy (n=71) versus those patients that did not get pregnant (n=101). The variables analysed were: women's age, KIDScore™, and the cellular timing division for 2 (t2), 3 (t3), 4 (t4), 5 (t5) cells and tB (time from insemination to blastocyst formation). Statistical analysis was performed with SPSS Version 20.0 statistical software package. Data were expressed as means±standard deviation (SD). Comparisons between groups were performed with Student's t-test. A value of *P*<0.05 was considered statistically significant.

**Results:** Comparing the non-pregnant group versus the pregnant group, the following results were observed, respectively: maternal age (35.8±4.1 *vs*. 35.0±3.7, *p*=0.198); Oocytes Aspirated mean (11.36±7.1 *vs*. 13.68±6.7, *p*=0.102); t2 (h) (26.82±3.52 *vs*. 26.49±3.61, *p*=0.551); t3(h) (37.15±4.05 *vs*. 37.03±4.46, *p*=0.857); t4(h) (38.69±3.99 *vs*. 38.76±4.60, *p*=0.908; t5 (h) (49.27±6.25 *vs*. 48.99±6,38, *p*=0.776); tB (h) (106.05±9.85 *vs*. 101.59±7,61, *p*=0.003); Dynamic Score Value, KIDScore™ (5.89±2.40 *vs*. 7.58, *p*=0.003).

**Conclusion:** It was observed that the tB was significantly less in patients that obtained pregnancy versus those that did not get pregnant. Conversely, the KIDScore™ was significantly higher in the pregnant group. The other measurements, t2, t3, t4, t5 that are part of the final KIDScore™, did not show any difference between groups. Therefore, is it possible that the difference in the final KIDScore™ for each group would be more related to the tB data, blastocyst expansion, and the inner cell mass and trophoblast scores, the last two also part of the KIDScore algorithm, than timing of cleavages.

**P-13. The impact of embryo morphology on pregnancy rate**

**Ricardo Marques de Azambuja** ^**1**^ **, Shana Wander Flach** ^**1**^ **, Marta Ribeiro Hentschke** ^**1**^ **, Natália Vasconcelos** ^**1**^ **, Mariangela Badalotti** ^**1**^ **, Alvaro Petracco** ^**1**^

^*1*^ *FERTILITAT - Centro de Medicina Reprodutiva.*

**Objective:** To evaluate if inner cell and trophoblast in the blastocyst morphology are associated with pregnancy rate .

**Methods:** A retrospective case-control study was performed, including 69 fresh single embryo transfer cycles. All the embryos were transferred in the blastocyst stage and were divided into two groups: implanted embryos (positive HCG) versus not implanted embryos (negative HCG). The variables analysed were: inner cell mass and trophoblast morphology classification according to Gardner blastocyst morphology (A, B or C). Statistical analysis was performed using SPSS Version 20.0 statistical software package. Data were expressed as a percentage. Comparisons between groups (A, B, and C) were performed with the Chi-Square test. A value of *p*<0.05 was considered statistically significant.

**Results:** A total of 69 embryos were evaluated: 34 in the positive hGC group and 35 in the negative hCG group. Analyzing the inner cell mass, the following results were found: in the hCG positive group, 68% of blastocysts were A (23/34), 26% were B (9/34), and 5% were C (2/34); in the negative hCG group, 46% were A (16/35), 37% were B (13/35), and 17% were C (6/35) without difference between groups (*p*=0.137). Analyzing the trophoblast, the following results were found: in the hCG positive group, 38% of blastocysts were A (13/34), 53% were B (18/34), and 9% were C (3/34); in the negative hCG group, 40% were A (14/35), 37% were B (13/35), and 23% were C (8/35), also without difference between groups (*p*=0.211).

**Conclusion:** Despite the improvement of the parameters for embryo morphology classification, it is still very difficult to identify embryos with the best chance of implantation based only on their morphology.

**P-14. Towards a perceptual metric for facial resemblance with application to gamete selection**

**Luis Arenaz Villalba** ^**1**^ **, Francho Meléndez** ^**1**^

^*1*^ *Software Engineering Fenomatch.*

**Objective:** When a gamete donor in an assisted reproduction process is needed, the aim is to find one that shares phenotypic traits such as eye color, skin, hair, or ethnicity with the patient. Clinics start to offer options that go further, aiming to find heritable characteristics through facial analysis.

**Methods:** We discuss here aspects of this analysis, its challenges, and the role of artificial intelligence. We focus on two questions: How can we measure facial resemblance between two people? And which of these facial features are heritable?.

**Results:**

*Facial Analysis*: The process of evaluating the resemblance between two people is a complex perceptual process extremely difficult to define in mathematical terms so it is developed into an algorithm. We summarize deep learning algorithms that can help in this process, highlighting the work on facial recognition and the generation of photorealistic faces.

*Facial Recognition*: Deep learning algorithms learn from data the characteristics needed to perform the given task. This puts the focus on the task, which makes the learned parameters optimal only to do this and not anything else. Take the case of differentiating identical twins; a facial recognition algorithm will optimize the characteristics that differentiate one from the other, like a scar or a tattoo, and it will give a high weight to those traits so it can distinguish a twin from its sibling. On the contrary, an algorithm focused on facial matching and heritable traits should ignore the specific differentiating peculiarities. Both algorithms need to describe a face mathematically, find common features, and distinguish people, however, they must be optimized for the particular task they are facing.

*Similarity*: The work in delves into the specific case of similarity between faces, and evaluates the differences between facial recognition and facial similarity, concluding that both tasks are similar, but not interchangeable and require different training methods and datasets. Perceptual metrics that seek to evaluate the similarity between visual stimuli in a similar way to that of a human being is also an active area of research in image processing.

*Heritability of facial features*: Anthropometric scientific research has confirmed that variation in human face morphology is driven by genetics. For example, moderate heritability has been found in the distances between nasion-basion and nasion-saddle, the position of the lower jaw and the nasal height. Dimosthenis Tsagkrasoulis et al. identified thousands of distances on the facial surface, using three-dimensional models, finding facial areas with high heritability. This study showed that facial complexity cannot be exclusively assessed by a small series of interfacial measurements.

*Kinship*: Verification of kinship from facial photographs has diverse applications from the automatic annotation of genealogical trees, to finding lost children or adoption. These algorithms seek to learn common traits in a family and how they are shared among family relationships. A metric that provides the probability of a donor to belong to the family of recipients would provide information on how facial features are transmitted and their heritability.

*Synthetic face generation*: The data that we use for the training of neural networks is crucial for the algorithm’s effectiveness. Data collection and annotation is arduous and limited by data protection. Generating data synthetically is extremely useful in these situations. Algorithms such as StyleGAN and automatic coding are capable of generating realistic high-quality faces that might be used for training.

**Conclusion:** Clinics increasingly make available to their patients tools that allow selecting the appropriate donor. Here we discuss areas in artificial intelligence which we believe are useful to address the analysis of phenotypic similarity of facial features, such as their heritability.

**P-15. Fertilization and embryo development of rescue in vitro maturation oocytes are not influenced by oocyte nuclear maturity stage**

**Marília Körbes Rockenbach** ^**1**^ **, Adriana Bos-Mikich** ^**2**^ **, Fernanda Kunrath Robin** ^**1**^ **, Nilo Frantz** ^**1**^ **, Gabriella Mamede Andrade** ^**1**^

^*1*^ *Nilo Frantz Reproductive Medicine - Porto Alegre, Rio Grande do Sul - Brazil.*

^*2*^ *Federal University of Rio Grande do Sul (UFRGS) - Porto Alegre, Rio Grande do Sul - Brazil.*

**Objective:** *In vitro* maturation of denuded immature oocytes (rescue IVM) may improve the efficiency of a cycle, especially in cases of a lower mature oocyte rate or poor responder patients. To better understand the potential of these immature oocytes, the aim of the study was to compare the fertilization and embryo development rates of in vivo matured oocytes (metaphase II - MII) with the in vitro matured metaphase I (MI) and germinal vesicle (GV).

**Methods:** In the present retrospective study, were included 78 stimulated cycles that were performed matured oocyte intracytoplasmic sperm injection (ICSI), at Nilo Frantz Reproductive Medicine between 2018 and 2021. The cumulus-oocyte complexes were denuded within 2-3 hours after oocyte recovery. MII oocytes were fertilized by ICSI and the immature ones (MI or GV) were cultured for approximately 24 additional hours, in CSCM-C (Continuous Single Culture Medium Complete). ICSI was performed in MI and GV oocytes that became MII with lab standard protocols. The fertilization was access after 18 hours and the embryo development in days 5, 6 and 7. The numbers of fertilized oocytes, cleaved embryos, morula and blastocyst were compared between in vivo and in vitro matured oocytes (MII vs matured MI, MII vs matured GV and matured MI vs matured GV). Statistical analysis were performed using GraphPad Prism 8.0^®^ and Kruskall-Wallis test was applied. *p*-value ≤ 0.05 was considered statistically significant. The results were presented as mean rank difference followed by *p*-value.

**Results:** In clinic routine, cycles of poor responders or with low rates of matured oocytes are candidate for rescue IVM as an attempt to improve outcomes. IVF cycles included in this study presented the average oocyte retrieval rates of 50.4%, 16.4% and 25.5% of MII, MI and GV, respectively. The rates of matured oocytes after rescue IVM was 75% for MI and 60.4% for GV oocytes. As expected, the fertilization rate was higher in the in vivo matured oocytes, when compared with MI (34.41; *p* = 0.0567) and GV (44.26; *p* = 0.0028) in vitro matured oocytes. Interesting, MI matured oocytes presented an increased rate of abnormal fertilization (>2PN) (12.96; *p* = 0.0218) and a lower number of embryos that cleaved (-30.38; *p* = 0.0265), compared with MII oocytes. However, no differences were observed considering the same parameters, when comparing MII and GV matured oocytes. Considering blastocyst development, MII oocytes had a greater outcome (35.54; *p* = 0.0077 and 35.23, *p* = 0.0046 for comparison between MI and GV matured oocytes, respectively).

**Conclusion:** As expected, fertilization and blastocyst rates were higher among the in vivo matured oocytes. Although fertilization, cleavage and blastocyst development did not differ between MI and GV *in vitro* matured oocytes, MI oocytes presented an increased rate of abnormal fertilization and a decreased proportion of cleavage stage embryos, when compared with MII oocytes. Considering that the MI oocytes are in a more advanced nuclear stage than GV oocytes and should complete nuclear maturation first, the prolonged culture period may be deleterious for the oocyte cytoplasmic components, key factors for an adequate early embryo developmental. Besides, it is important to mention that no media supplements or specific IVM protocol were applied in these IVM procedures. Rescue IVM represents an attempt to improve outcomes in poor cycles and should be considered for particular cases, despite the low rates of blastocyst formation.

**P-16. The quality of life of women undergoing infertility treatment prior and in the beginning of the COVID-19 pandemic**

**Fernanda Polisseni** ^**1**^ **, Laura de Souza Bechara Secchin** ^**1**^ **, Refaela Heloisa de Almeida** ^**1**^ **, Larissa Milani Coutinho** ^**1**^ **, Juliana Polisseni Magalhaes** ^**1**^ **, Joselio Vitoi Rosa** ^**1**^

^*1*^ *Clínica Nidus Medicina Reprodutiva.*

**Objective:** Both the treatment of infertility and the infertility itself may significantly affect the patient’s overall sense of wellbeing. The present study aimed to measure the quality of life (QoL) among infertile women and identify the factors related to good or poor QoL in this group, prior and in the beginning of the Covid-19 pandemic.

**Methods:** All of the 205 infertile women seen at a Brazilian private Reproductive Medicine clinic, from October 2019 to April 2020, received an online formulary containing a sociodemographic questionnaire and the FertiQol, a specific screening self-report instrument for analyzing QoL in infertile patients. After signing the free and informed consent term, the patients were able to complete, anonymously and voluntarily, the 11 structured sociodemographic questions, with a specific query concerning the impact of COVID-19 pandemic in the infertility treatment. The FertiQol consists of 24 items regarding the influence of fertility problems in the emotional, mind/body, relationship and social domains (Core FertiQol); 10 treatment-related items; and 2 items concerning patients’ self-perception of their overall QoL and physical health.

**Results:** Out of the 205 eligible patients, 71 have consented to participate in the study and have filled out the online questionnaire completely. The patients’ mean age was 35.41±0.56, ranging between 21 and 45 years old. The average time of infertility was 3.59±2.62 years. Regarding educational level, 22.5% of the patients had a middle school education, 21.13% had a university degree and 45.0% had a postgraduate degree. About 47.89% of the participants reported that the COVID-19 pandemic had a great or a complete impact in their QoL, concerning the infertility treatment. Multiple regression analysis was used to verify whether sociodemographic variables could interfere in the QoL of patients with infertility. Regarding the Core FertiQoL (n=71), a linear association between educational level and QoL scores was identified in the emotional (β=0,330), mind/body (β=0,273), and social (β=0,288) subscales. The negative impact of the COVID-19 pandemic in the infertility treatment was shown as a predictor factor for lower scores of QoL in the emotional (β=-0,238) and mind/body (β=-0,356) subscales. Considering the Treatment FertiQol module (n=63), the pandemic impact on infertility treatments was considered a predictor factor (β=-0,272) for worse QoL in the Treatment Tolerability subscale. Finally, the multiple linear regression showed that both the educational level (β=0,317) and the impact of COVID-19 pandemic in patients’ treatments (β=-0,317) are predictors for the Total FertiQol scores (n=71).

**Conclusion:** Higher educational levels were associated with better QoL scores among infertile patients. The COVID-19 pandemic was associated with a negative impact on the QoL of these women since the first months of the pandemic, manly with regard to the Treatment Tolerability subscale.

**P-17. Marital infertility: Issues related to quality of life, anxiety and spiritual well-being in patients from the Assisted Reproduction program at a University Hospital**

**Ana Karoline Machado da Rosa** ^**1**^ **, Laiza Simone Garcia Quadro** ^**1**^ **, Andressa da Silva Behenck** ^**1**^ **, Suzana de Azevedo Zachia** ^**1**^ **, Flavia Sarvacinski** ^**1**^ **, Cristina Palma Kuhl** ^**1**^ **, Markus Berger** ^**1**^ **, Isabel Cirne Lima de Oliveira Durli** ^**1**^ **, Eduardo Pandolfi Passos** ^**1**^ **, Paula Barros Terraciano** ^**1**^

^*1*^ *Hospital de Clínicas de Porto Alegre.*

**Objective:** To assess whether female infertility impairs the quality of life of women and their partners.

**Methods:** The study was guided by a descriptive observational cross-sectional design. Data from 59 couples, aged 25 to 40 years, all enrolled in the assisted reproduction program of a university hospital, were analyzed. The instruments used were: World Health Organization Quality of Life, BREF version (WHOQOL-BREF), Beck Anxiety Inventory (BAI), Spiritual Well-Being Scale (EBE) and sociodemographic questionnaire, by signing the Informed Consent Term.

**Results:** Studies associate marital infertility as a cause of numerous emotional disorders. However, the results found after applying the BAI, identified levels of anxiety considered low, at most 06 points among the participants, with no significant difference between women and men, possibly because patients received ample emotional support during the period they were in the COVID-19 epidemic. The information of an extremely anxious group was not verified, even when they were related to EBE and WHOQOL-BREF. Furthermore, it was noteworthy that women who do not have a religion have a higher performance in the physical domain of the WHOQoL BREF (64.3%) compared to those who follow some creed. Also, it is observed that women who have children produce lower performance in the physical domain of the BREF, the demands of motherhood may be implied in this case. Regarding QoL associations, men who live with other people and/or family members besides their wife have better QoL scores in the physical and psychological domains compared to those who only live with their wife, which corroborates the benefits of social support. Concerning remuneration, men who earn more also have better scores in the psychological and environmental domains. The cause of infertility was a significant predictor of anxiety and distress in our sample, with higher levels in cases of female infertility, this fact is probably related to the feeling of guilt and broken expectations.

**Conclusion:** Frustration was present in both men and women. Most people will face a dilemma, exposed to stigmatization and self-blame, which requires a differentiated and attentive look at each patient with complementary practices and therapies. Seeking to increase the quality of life of these patients assisted by the service, so that, as a couple, they are able to proceed with the fertilization program.

**P-18. The impact of the exposure times to equilibration solution during vitrification on the lipid profile of mice oocytes**

**Thalita S. Berteli** ^**1**^ **, Eduardo D. Borges** ^**1**^ **, Caroline M. Da Luz** ^**1**^ **, Paula A. Navarro** ^**1**^ **, Alessandra A. Vireque** ^**2**^

^*1*^ *Faculdade de Medicina de Ribeirão Preto, Universidade de São Paulo - Departamento de Ginecologia e Obstetrícia, Ribeirão Preto - SP, Brazil.*

^*2*^ *Invitra - Assisted Reproductive Technologies LTD, Supera Innovation and Technology Park, Ribeirão Preto - SP, Brazil.*

**Objective:** In order to obtain a stable glassy state in the vitrification process, it is necessary to combine high cooling rates, small volumes of highly concentrated cryoprotectant solutions and short exposure times. During equilibration phase of oocyte vitrification, the exposure time varies from 10 to 15 minutes according to manufacturers’ recommendations and a dynamic flow of water and solutes occurs through plasma membrane, a step closely related to oocyte quality maintenance after warming. Studies investigating oocyte lipid profile, especially lipids related to membrane lipid bilayer, may be useful to evaluate the impact of the equilibration protocol of vitrification on oocyte cryotolerance. Here we report the feasibility of the targeted lipidomics using multiple reaction monitor profiling (MRM-profiling) mass spectrometry to investigate and monitor the impact of the exposure times to equilibration solution (ES) during vitrification on lipid profile of mice oocytes.

**Method:** Experimental study including C57BL/6J mice. Oocytes (3 replicates; n=20 oocytes/group) were collected after superovulation with eCG and hCG and exposed to ES (Irvine Scientific) for distinct durations. The two groups were vitrified following the manufacturer recommended protocol: 10-minute total duration (oocytes stay for 6 minutes in third drop - ES10) or a shorter, 7-minute total protocol (oocytes stay for 3 minutes in the third drop - ES7). At the final step of equilibration, oocytes were washed 3 times in methanol: H2O solution (1:3 v/v) and kept at -80o C until lipid extraction. Lipids, equivalent to a pool of 5-7 oocytes per group/replicate were extracted using One Step Methanol protocol and flow injected into the triple quadrupole spectrometer equipped with an electrospray ion source (ESI-MS). Lipid classes were analyzed by MRM-profiling. The relative ion intensities values were evaluated using univariate (change fold, t-test, volcano plot), and multivariate analysis (PCA, PLS-DA, cluster analysis). Most informative lipid species in each group were sorted out using the partial least square discriminant analysis (PLS-DA) variables of importance (VIP) scores >1.

**Results:** Higher abundances for the saturated free fatty acids C19:0, C20:0 and C22:0 were observed in ES10 (change fold>2; *p*<0.05) when compared to ES7 group. In the PCA scores, ES7 and ES10- CTR oocytes showed a tendency of discrimination at PC1 (68,8% of variability explained) and 4 membrane phospholipids (phosphatidylcholines and sphingomyelins) classified by PLS-DA were downregulated in ES10, indicating the presence of lipid composition changes in mice oocyte membrane due to amount of equilibration time used for the vitrification protocol.

**Conclusion:** Minimal changes in vitrification protocol such as the duration of the equilibration phase were observed in the lipid profile of mice oocytes. Our results indicate that shorter time exposure in ES compared to long equilibration recommended by the manufacturer (7 min versus 10 min, respectively) is related to less disturbance of the plasma membrane of mice oocytes.

**Supported by:** Coordination for the Improvement of Higher Education Personnel (CAPES, Finance Code: 001, process number 88887.371487/2019-00, and process n. 88887.597054/2021-00); Foundation to Support Teaching, Research, and Assistance (FAEPA) at Clinical Hospital, Faculty of Medicine of Ribeirão Preto, University of São Paulo; Invitra Assisted Reproductive Technologies LTDA; andBrazilian National Council for Scientific and Technological Development (CNPq), (process n. 305173/2019-7).

**P-19. Effect of C-type natriuretic peptide supplementation on in vitro culture of bovine embryos**

**Isabella Maran Pereira** ^**1**^ **, Camila Bortoliero Costa** ^**1,2,3**^ **, Elisa Mariano Pioltine** ^**1,2**^ **, Marcelo Marcondes Seneda** ^**3**^ **, Marcelo Fábio Gouveia Nogueira** ^**1,2**^

^*1*^ *Laboratory of Embryonic Manipulation, School of Sciences and Languages, São Paulo State University (UNESP), Assis, SP, Brazil.*

^*2*^ *Graduate Program in Pharmacology and Biotechnology, Institute of Biosciences, UNESP, Botucatu, SP, Brazil.*

^*3*^ *Laboratory of Animal Reproduction, University of Londrina (UEL), Londrina, PR, Brazil.*

**Objective:** *In vitro* produced (IVP) embryos are reported to have low resistance to cryopreservation which, apparently, is associated with the lipid content, as well as the composition and morphology of the organelles present in the embryonic cells. Aiming at the reduction of the lipid content, several molecules have already been tested, with the modulators of cyclic adenosine monophosphate (cAMP) being associated with the reduction of intracytoplasmic lipid droplets. In this way, the C-type natriuretic peptide (CNP) stands out as a cAMP modulator molecule. It is well known its action on the maintenance of meiotic arrest in oocytes from different species of mammals. However, the relationship between CNP and the embryo has been poorly reported. Thus, this study aimed to evaluate the effect of two concentrations of CNP (Control; 100 nM - Group CNP-100; 400 nM - Group CNP-400) since the first day (D1) of in vitro culture with high oxygen tension (20%), on bovine (Bos taurus indicus) blastocyst and hatching rates.

**Methods:** The evaluations were carried out on days 7, 8 and 9 after the fertilization step. Five replicates were performed with approximately 100 presumptive zygotes/group. Data were tested about their normal distribution and homogeneity of variance through the Shapiro-Wilk test. The Gaussian data were then submitted to one-way ANOVA.

**Results:** Both the blastocyst and the hatching rates did not differ from the control group (p=0.84 and p=0.86, respectively), as the blastocyst rate for CNP-100 (33.39 ± 3.19) and CNP-400 (33.89 ± 1.71) were similar to the Control (34.08 ± 7.73). The hatching rates were 43.80 ± 14.61, 44.80 ± 12.73 and 45.40 ± 16.47, respectively for CNP-100, CNP-400, and Control. I.

**Conclusion:** the supplementation of CNP in in vitro culture of bovine embryos did not significantly alter the embryo production rate. It is an indication of absence of embryotoxicity even at the highest concentration (showing that 400nM concentration can be used in future studies). A study in progress is searching to clarify the embryo-CNP interaction and to investigate an eventual changing in the embryonic lipid profile.

**P-20. Telomere length increases and line-1 copy number decreases with aging in human sperm**

**Thalita S. Berteli** ^**1,2**^ **, Fang Wang** ^**1**^ **, Paula A. Navarro** ^**2**^ **, Fabiana B. Kohlrausch** ^**1,3**^ **, David Lawrence Keefe** ^**1**^

^*1*^ *New York University, Langone Medical Center, New York, NY, Estados Unidos da América.*

^*2*^ *Departamento de Ginecologia e Obstetrícia, Faculdade de Medicina de Ribeirão Preto, Universidade de São Paulo (FMRP-USP), Ribeirão Preto, São Paulo, Brasi* ^*l*^ *.*

^*3*^ *Programa de Pós Graduação em Ciências e Biotecnologia, Universidade Federal Fluminense (UFF), Niterói, RJ, Brasil.*

**Objective:** Unlike other cells in the body, in sperm, telomere length (TL) increases with age. TL can regulate nearby genes, and the sub telomeric region is rich in retrotransposons, so we hypothesized that age-related telomere lengthening in sperm may suppress LINE-1 (L1), the only competent retrotransposon in humans. We measured L1 copy number (L1-CN) and TL in sperm (STL) from young and older men to evaluate the relationship between paternal age, TL and L1-CN. We also evaluated L1-CN and TL in individual sperm to determine whether these variables influence sperm morphology.

**Methods:** Prospective case-control pilot study including sperm samples (n=36) from men, aged 29-55, undergoing infertility treatment at NYU Langone Fertility Center. To compare L1-CN and TL in normal and abnormal morphology single sperm, sperm samples (n=7), aged 30-48, also were included. Twenty individual sperm (10 normal and 10 abnormal) from each man were selected. STL was assayed by mmqPCR and L1-CN by qPCR. To verify the relationship between age and STL or L1-CN, Pearson's correlation coefficient was performed. To verify whether there is a statistical difference between STL and the stratified variable age (≤40 years and ≥45 years), the Wilcoxon nonparametric test for independent samples was applied. To analyze if there is a statistical difference between sperm morphology (normal and abnormal group) and STL or L1-CN, the Wilcoxon nonparametric test was applied for independent samples. Analyzes were implemented in the SAS version 9.4 program. A *p*-value <0.05 was considered significant.

**Results:** The median age of the 36 ejaculated samples included in this study was 41 (interquartile range - IQR; 36-44) years old, the volume was 2.6 (1.70-3.95) ml, the concentration of spermatozoa per ml was 77 (40.50-96.50), and the percentage of progressive motility was 56.5 (50-65). STL was positively correlated with age (n: 36; r = 0.4096; p=0.0131). STL from older (age ≥45 years, n: 7; Median, IQR: 1.55, 1.46-2.11) was significantly longer than younger men (age ≤40 years, n: 16, Median, IQR: 1.05, 0.96-1.21) (*p*=0.008). L1 copy number in sperm decreased significantly with paternal age (n: 36; r = -0.3443; *p*=0.0397), being significantly lower in older (age ≥ 45 years, n: 7; Median, IQR: 0.81, 0.75-0.89) compared to younger men (≤ 40 years, n: 16, Median, IQR: 1.00, 0.94-1.09) (*p*=0.0029). From (n: 7) samples included in single cell experiment, the median (IQR) age was 41 (36-47) years old. The volume of the samples included was 3.0 (2.0-4.3) ml. The concentration of spermatozoa per ml was 60 (20-99), and the percentage of progressive motility was 52 (50-65). STL in normal morphology single sperm (n: 49; Median, IQR: 8.55, 6.09-21.95) was significantly higher than abnormal sperm (n: 58, Median, IQR: 4.89, 2.74-8.97) (*p*=0.0015). L1 copy number did not differ between normal (n: 49, Median, IQR: 1.84, 1.01-3.05) and abnormal sperm (n: 58, Median, IQR: 1.80, 1.01-2.94) (*p*=0.7239).

**Conclusion:** L1-CN decreases significantly with paternal age, as sperm telomeres lengthen. Furthermore, morphologically normal sperm has longer telomeres than abnormal sperm. Telomere elongation in the male germ line may serve to repress retrotransposition, which tends to increase with cellular aging. Further studies in larger cohorts over a wide age span are needed to confirm our conclusions and explore their biological and clinical significance.

**Supported by:** Coordination for the Improvement of Higher Education Personnel (CAPES, Finance Code: 001, Brazil; Grant number 88887.371487/2019-00), Brazilian National Council for Scientific and Technological Development (CNPq, Brazil; Grant number 204747/2018-0) and the Stanley H. Kaplan Fund of the NYU.

**P-21. Oocyte cryopreservation is a valuable and safe ART to women who aim to postpone maternity**

**Carolina Lumertz Martello** ^**1**^ **, Paula Flores Ternus** ^**1**^ **, Adriana Bos-Mikich** ^**2**^ **, Nilo Frantz** ^**1**^

^*1*^ *Nilo Frantz Reproductive Medicine, Porto Alegre, RS, Brazil.*

^*2*^ *Basic Health Sciences Institute, Federal University of Rio Grande do Sul, Porto Alegre, RS, Brazil.*

**Objective:** The desire for fertility preservation and maternity postponing has gradually increased oocyte cryopreservation adherence. However, does the oocyte vitrification and warming technique impair the in vitro fertilization (IVF) cycle outcome? This work aims to evaluate and compare the outcomes between vitrified and fresh autologous oocyte cycles in a Brazilian human reproduction center.

**Methods:** Retrospective study including 39 IVF cycles with vitrified and warmed oocytes (vitrified/warmed oocytes group). The control group of cycles using fresh oocytes (fresh oocytes group) was selected considering the primary infertility factor and biopsy or embryo transfer. Groups were compared firstly by age, anti-Müllerian hormone (AMH), and body mass index (BMI). Secondly, the number of inseminated oocytes, fertilization rates, transferred embryos rates, and vitrified/biopsied embryos rates were compared. Finally, pregnancy, ongoing/delivery, and miscarriage rates were analyzed. From the embryo biopsy cycles, the euploid rates were compared. Moreover, a small group of cycles using sibling oocytes (vitrified/warmed and fresh oocytes in the same cycle) were separately analyzed (sibling oocytes group, n = 7). Comparisons were performed using unpaired Student’s *t*-test and Fisher’s exact test when appropriated using GraphPad Prism 6 and considering significant *p* value ≤0.05.

**Results:** Overall, baseline characteristics such as age and BMI were similar between groups. Interestingly, the vitrified/warmed oocyte group presented a higher mean AMH when compared to the fresh oocytes group (*p*=0.0091). Laboratory outcomes (inseminated oocytes, transferred embryos, and vitrified/biopsied embryos) were similar between groups. Fertilization rate was higher in the fresh oocyte group when compared to the vitrified/warmed oocyte, 76.1% and 66.3%, respectively (*p*=0.0078). Moreover, euploid rates were similar between vitrified/warmed oocytes and fresh ones. Regarding clinical outcomes, pregnancy, ongoing/delivery, and miscarriage rates were similar between the two groups. Fertility preservation patients waited an average of 3.5 years to perform oocytes warming and IVF after the cryopreservation. The sibling oocytes groups presented similarity in all parameters analyzed.

**Conclusion:** Women seeking to preserve their fertility in the vitrified/warmed oocyte group presented a higher AMH as they usually do not have infertility factors. Although the data showed a reduction in fertilization efficiency due to the vitrification and warming process, this effect had no impact on embryo development. Vitrification and warming oocyte techniques do not change clinical IVF treatment outcomes. Therefore, vitrification of oocytes seems a valuable and safe option to women who aim to postpone maternity.

**P-22. Female Body Mass Index and its impact on clinical e neonatal outcomes of FET cycles**

**VIctoria Campos Dornelles** ^**1**^ **, Isadora Badalotti-Teloken** ^**1**^ **, Marta Ribeiro Hentschke** ^**1**^ **, Natalia Fontoura Vasconcelos** ^**1**^ **, Alvaro Petracco** ^**1**^ **, Mariangela Badalotti** ^**1**^

^*1*^ *Fertilitat - Centro de Medicina Reprodutiva.*

**Objective:** To evaluate the impact of female body mass index (BMI) on frozen embryo transfer (FET) cycles of assisted reproduction techniques (ART).

**Methods:** Retrospective cohort study performed at an assisted reproductive clinic in southern Brazil, with data collected from electronic records from 2013 to 2020. A total of 347 FET cycles were included. Sample was divided into 3 groups according to female BMI: Group 1, normal BMI (n= 244), Group 2, overweight (n= 195), and Group 3, obese (n= 38). Variables were compared between groups and were expressed as mean±SD or n(%). ANOVA, T-test and X2 were applied. Statistical significance was defined as *p*<0.05.

**Results:** When comparing G1 vs. G2 vs. G3, the following results were found, respectively: maternal age (35.9±4 *vs*. 36±4.8 *vs*. 37.3±4.3, *p*=0.183); clinical pregnancy rates per cycle (43.1% *vs*. 37.7% *vs*. 28.9%, *p*=0.379); macrosomic newborns (2.2% *vs*. 6.7% *vs*. 28.6%, *p*=0.086); low weight newborns (24.4% *vs*. 26.7% *vs*. 14.3%, *p*=0.990); normal weight newborns (73.3% *vs*. 66.7% *vs*. 57.1%, *p*= 0.579); preterm newborns (26.7% *vs*. 20% *vs*. 14.3%, *p*=0.978). When comparing G1 *vs*. G3, the implantation rate was 33.4% *vs*. 25%, respectively, *p*=0.408 .

**Conclusion:** In this study, overweight and obesity were not associated with worst FET clinical outcomes; however, there was a tendency of worst clinical pregnancy rates and worst implantation rates the higher was the maternal BMI. This difference, despite not having statistical significance, is clinically relevant for each patient seeking motherhood with ART. Thus, there seemed to have more macrosomic newborns in the obese group. More studies including larger samples of FET cycles should be performed for further conclusions, as the most available studies regarding ART and BMI only analyze fresh cycles.

**P-23. Usage of microfluidic device for sperm selection and correlation with outcomes of *in vitro* fertilization cycles and pregnancy rates**

**Tayara de Moraes Vergueiro Tavares** ^**1**^ **, Gustavo Nardini Cecchino** ^**2**^ **, Erika Oliveira Semaco Tamaru** ^**1**^ **, Rafael Portela** ^**1**^ **, Laura Maria Freitas Silva** ^**1**^ **, Matheus Teixeira Roque** ^**2**^ **, Rodrigo da Rosa Filho** ^**2**^ **, José Geraldo Aguiar Faria Junior** ^**1**^ **, Philip Wolff** ^**1**^

^*1*^ *URO-USP - Dpto de Urologia da Faculdade de Medicina de São Paulo, São Paulo - SP.*

^*2*^ *Patologia-USP - Dept. de Patologia da Faculdade de Medicina de São Paulo, São Paulo - SP.*

**Objective:** It is well-known that high levels of sperm DNA fragmentation negatively impact not only early embryonic development but also embryo genome activation. Microfluidic technologies for processing sperm samples selection such as the ZyMōtTM device have been developed to sort sperm with lower rates of sperm DNA fragmentation in order to improve in vitro fertilization (IVF) outcomes. However, the real benefits on the clinical and laboratory outcomes of IVF cycles are yet to be determined. The aim of this study was to demonstrate if sperm selection by a microfluidic sperm-sorting device improves laboratory outcomes of intracytoplasmic sperm injection cycles and pregnancy rates.

**Methods:** We performed a retrospective study including 126 seminal samples processed between May 2019 and December 2020. These samples belong to 63 infertile couples that underwent 2 consecutive IVF cycles using the swim-up technique in the first cycle (n=63) and the ZyMōtTM device in the second (n=63). A total of 118 seminal samples from 59 couples were included. Four couples were excluded due to insufficient seminal volume / concentration (2) or absence of mature oocytes recovered (2). Laboratory outcomes were evaluated including semen parameters post processing, as well as fertilization, cleavage, blastulation (blastocyst among cleavage stage) and top-quality blastocyst rates. Continuous variables were analyzed by paired Student’s t-test or Wilcoxon signed-rank test, as appropriate. Differences were considered significant when *p*-value <0.05.

**Results:** We did not find any difference in the percentage of non-progressive sperm motility [0 (0-33%) *vs*. 0 (0-10%); *p*=0.063] and immotile sperm [0 (0-70%) *vs*. 0 (0-80%); *p*=0.095] post processing when comparing the swim-up technique with the ZyMōtTM device. Likewise, no significant differences were detected regarding fertilization [83% (0-100%) *vs*. 89% (0-100%); *p*=0.104] and cleavage [100% (0-100%) *vs*. 100% (80-100%); *p*=0.217] rates in both groups. Nevertheless, there was a significant difference in the percentage of progressive sperm motility [100% (20-100%) *vs*. 100% (10-100%); *p*=0.012] post processing, as well as blastulation [37%±0.32 *vs*. 50%±0.3; *p*=0.031] and top-quality blastocyst [13.5% (0-100%) *vs*. 33% (0-100%); *p*=0.009] rates, favoring the ZyMōtTM device. Ultimately, no difference was found in the pregnancy rates [70 (7/10) *vs*. 64,2% (9/14); *p*=0.69].

**Conclusion:** Besides the ZyMōtTM device has been indicated as a new tool to potentially improve laboratory and clinical IVF outcomes, it offers the advantage of being safe, fast, easy-to-use and less influenced by human errors. Further well-designed prospective studies are needed to prove its cost-effectiveness, including analyses of a higher number of IVF cycles.

**P-24. Oocyte donation: a comparative study of the new Brazilian resolution with other countries' regulations**

**Marina Galego Teixeira1, Gabriel Monteiro Pinheiro1, Isadora Germano Fernandes Bastos1, Karolyne Vale de Sá1**

^*1*^ *Universidade Santo Amaro.*

**Objective:** To analyze the new ethical standards of the 2021 Federal Council of Medicine Resolution on oocyte donation and to make a comparison with the regulations of different countries.

**Method:** An active search was conducted in SciELO and Pubmed databases, and resolutions of the National Health Surveillance Agency (ANVISA), the Federal Council of Medicine (CFM) and the European Society of Human Reproduction and Embryology (ESHRE).

**Results:** The donation of gametes and embryos in Brazil is regulated by CFM resolution 2,294, published on May 27, 2021. According to the ethical standards, donation cannot be of a lucrative or commercial nature and donors should not know the identity of the recipients and vice-versa, except when gamete donation occurs between relatives up to the 4th degree. The age limit for egg donation is 37 years. Besides solidary donation, shared oocyte donation is also possible and occurs when donor and recipient share both the gametes and the financial costs involved in the in vitro fertilization procedure. Regarding the donor's physical condition, in Brazil, women up to 37 years old are allowed to donate their oocytes, in order to prioritize the oocyte quality. In France, United Kingdom, United States of America (USA) and Portugal, the ages considered appropriate for donations are very close to Brazil's, with France being the closest, at 38, and Portugal the most restrictive, accepting egg donation from women up to 33. The recognition of the identity between donors and recipients, in Brazil, as well as in Spain, Portugal and France, is not allowed, ensuring the donor's anonymity, unlike what happens in the UK, where the children, in certain age groups, have the right to access the donor's identity. In the US, the donor can choose to be identified or not. The literature shows that the effect of anonymity on donor volume remains controversial, and may or may not be a stimulus. Despite the ethical clash between altruism as a primary or secondary role in gamete donation, in countries where egg donation cannot be profitable or commercial, there is a shortage of egg donation, making waiting lists for a donated egg long. In countries like UK and USA, the financial compensation of donors is allowed. In France, the health system itself finances the assisted reproduction treatment of citizens until the age of 43, and, as in Brazil, gamete donation does not aim at any lucrative purpose. In Spain, even if the donation is defined as altruistic, financial compensation is allowed for the donors for the time and effort involved in the process. In Portugal, the law provides that, financially, there is only compensation for any expenses involved in the process. ESHRE explains that due to the different legislations in the European Union countries, it is recommended that donors be informed about the ethical debate of payment and compensation. Meanwhile, in the US, according to the American Society for Assisted Reproduction (ASRM), payment for donation is justified, but it is recommended to limit the number of donations in order to prevail altruism.

**Conclusion:** Although Assisted Reproduction techniques are increasingly present in family planning, it is observed that the practice of ovodonation is directly linked to each country's culture and regulations. Although the norms used in Brazil have convergence with those of many countries, it is worth emphasizing the need for more stimulating guidelines for oocyte donation, as well as the guarantee that all processes involved can occur in an ethical and standardized manner.

**P-25. Monozygotic twinning in ART cycles: which are the roles of zygotic polarity and genetics?**

**Adriana Bos-Mikich** ^**1**^ **, Carolina Lumertz Martello** ^**2**^ **, Gabriella Mamede Andrade** ^**2**^ **, Nilo Frantz** ^**2**^

^*1*^ *Universidade Federal do Rio Grande do Sul.*

^*2*^ *Nilo Frantz Medicina Reprodutiva.*

**Objective:** The incidence of monozygotic twining (MZT) is increased after ART procedures. However, little is known about the mechanisms behind the MZT associated with IVF. Zygotic non-polarization is known to lead to anomalies in development. Also, the role of a genetic contribution shall not be dismissed. The objective of the present study is to assess the possible contribution of zygotic polarity and genetic factors to MZT gestations after IVF in a single assisted reproduction center.

**Methods:** Retrospective analyze of 22 zygote images, from 16 frozen embryo transfers (10 single, six double blastocyst transfers), that resulted in MZT gestations between 2017 and 2020. The pattern of alignment of the PNs toward the polar bodies (PBs), described as zygotic polarity, Z- score and the presence of cytoplasmic halo were recorded between 18 and 19h post-insemination by ICSI. Electronic questionnaire was sent to the 14 couples that had MZT gestations on the occurrence of multiples pregnancies in their families. Two couples received oocytes from egg donation. MZT was assessed by ultrasound at 6-7 and 8-9 weeks of gestation.

**Results:** Ten single embryo transfers resulted in nine twin (seven 2GS\2 embryos; two 1GS\2 embryos) and one triplet (3GS\3 embryos) gestations. Five sets of twins (six girls and four boys) and one set of triplets (three boys) were born. Four twin gestations ended in abortions. Five double embryo transfers resulted in one set of twins (1GS, two girls), one set of triplets (2GS, two girls and one boy) and one single baby (3GS, 1 boy) born. One twin and one triplet pregnancies ended in abortions. At the check point analyzed, none of the zygotes presented their PN aligned with the PBs. The majority of the zygotes presented poor Z-score (Z2-3\4;14 out of 20, one was not scored). Cytoplasmic halo was present in all zygotes, being that in five instances, it was clearly evident even between 18 and 19hrs post-insemination. Two pregnancies resulted from oocyte donation from the same egg donor, one fresh cycle and one cryopreserved oocytes. Both egg recipients received one blastocyst, one resulted in triplets and the other in twins. Nine out of 14 couples with autologous oocytes answered the questionnaire on the occurrence of twinning in their families. Four couples declared the incidence of monozygotic twinning (40.4%) and one declared three sets of dizygotic twins among their relatives.

**Conclusion:** The genetic background of MZT in ART has not received much attention. Our data reinforces the idea of an autosomal dominant genetic component in MZT. It is possible that the expression of genes involved in monozygotic twinning in specific families is enhanced by ART due to ovarian stimulation, trigger, insemination techniques, zona pellucida breach and overall lab environmental exposure and micromanipulation. The lack of zygotic polarity and delayed cytoplasmic events may represent important developmental factors, that further facilitate the generation of MZT in these families. Thus, considering the higher risk of mortality and complications that can arise in monozygotic multiple gestations, collection of detailed familial information is paramount for couples undergoing ART procedures and for oocyte donor recruitment.

**P-26. The impact of covid-19 on oocyte storage for elective fertility preservation: patients’ profile and cycle characteristics**

**Adriana Bos-Mikich** ^**1**^ **, Luiza Mezzomo Donatti** ^**2**^ **, Gabriella Mamede Andrade** ^**2**^ **, Nilo Frantz** ^**2**^

^*1*^ *Universidade Federal do Rio Grande do Sul.*

^*2*^ *Nilo Frantz Medicina Reprodutiva.*

**Objective:** Data collection and comparative report on the impact of covid-19 pandemic on oocyte cryopreservation cycles in a single reproductive center. The report aims to describe the patients’ profile and their cycle characteristics, considering two groups of women, oncological patients and general female population, in two distinctive periods.

**Methods:** Retrospective analysis of 90 (2018-2019, period 1) and 99 (2020-2021, period 2) cycles of oocyte cryopreservation, before and after the beginning of covid-19 pandemic. Parameters analyzed were women’s occupation, age, number of follicles recruited, number of oocytes collected, number of MII oocytes cryopreserved, days of ovarian stimulation and anti-Mullerian hormone (AMH). Two-way ANOVA test was applied to examine the effect of the different variables.

**Results:** Considering that period 2 represents 20 months, not two full years, there was an increase in the overall number of cycles during the pandemic months compared to period 1. However, despite the low numbers in the period before (n=9) and after covid-19 (n=5), we observed a decrease in oncological patients seeking oocyte cryostorage during the pandemic months. The vast majority of oocyte cryopreservation cycles (93%) were performed by women that wished to postpone pregnancy for personal reasons. Along the periods 1 and 2, most women of the general population belonged to the 35-39 years age group (64% and 71%, respectively), followed by 30-34 years (22% and 28%, respectively) and only two and one at the age of 25-29 years, respectively. Cancer patients were distributed along the different age groups being that the majority was younger than 35 years, in periods 1 and 2 (n=6/9 and n=3/5, respectively). Majority of women from the general population had a university degree and underwent one cycle of oocyte cryostorage, 20 had two and one had three cycles. The oncological population was represented by 11 women with higher education and one student (78% and 100% of responders, in period 1 and 2, respectively) and all of them had one cycle of gamete cryostorage. There were no statistically significant differences in the mean values for women age, number of follicles, oocytes collected, MII cryopreserved, days of ovarian stimulation and AMH level, when comparing the general population and oncological patients, between period 1 and 2. Seven cycles in the general population and one in the oncological group had no MII oocyte for storage. Finally, we observed that less than half (42%) of the general female population has enough oocytes stored (>8 gametes) to achieve success in terms of a baby born. Among the cancer patients, 57% of them reached the minimum number of cryostored oocytes or more, to have a reasonable chance of success.

**Conclusion:** Women that are motivated to postpone childbearing by age should be counselled to seek for help at a younger age by public awareness campaigns. The decrease in oncological patients during pandemics may represent a detrimental impact of covid-19 pandemic on the usual care in many cancer care facilities. Both groups of patients should be clearly informed about their future possibilities of having a biological child, by specialists involved in fertility preservation care.

**P-27. Shared oocyte donation program: a 15-years experience**

**Talita Colombo, Marta Hentschke** ^**1**^ **, Victoria Dornelles** ^**1**^ **, Ariane Kira** ^**1**^ **, Vanessa Trindade** ^**1**^ **, Isadora Badalotti-Teloken** ^**1**^ **, Debora Farinati** ^**1**^ **, Maria Tereza Sanseverino** ^**1**^ **, Alvaro Petracco** ^**1**^ **, Mariangela Badalotti** ^**1**^ **, Adriana Arent** ^**1**^

^*1*^ *Fertilitat - Medicine Reproductive Center - Porto Alegre - RS.*

**Introduction:** To evaluate assisted reproductive outcomes of patients participating in a shared egg donation program.

**Methods:** A retrospective cohort study, performed at a private clinic in Southern Brazil, between Jan/2002 to Dec/2017. The study included 586 fresh shared oocyte donation cycles (290 cycles from donors and 296 cycles from recipients), resulting in 473 fresh embryo transfers. The egg division was equally made, whereas, at an odd amount, the donor always had preference. The data were collected from an electronic database. The statistical analyses included Chi-square test, Fisher's exact test, Mann-Whitney U-test or Student t-test depending on the data distribution, associated with multivariate logistic regression, and considering significant *p*<0.05.

**Results:** Comparing Donors and Recipients, the following results were found: Women Age (30.6±3.3 *vs*. 43.4±4.9, *p*<000.1); Median number of inseminated oocyte [6 (4-8) *vs*. 5 (3-7)] Fertilization rates (72.0±21.4 *vs*. 74.6±24.2, *p*<0,001); Number of D3 good quality embryos [2(2-3) *vs*. 2(2-4); *p*=0.446] and number of blastocyst [3(2-4) *vs*. 3(2-4), *p*=0.128]. Also, Implantation rate, % (46.2 *vs*. 48.5, *p*=0.67), clinical pregnancy rate, % (41.9 *vs*. 37.7, *p*=0.39), live birth rates per transfer, % (34.2 *vs*. 29.1, *p*=0.23), miscarriage rate (16.3 *vs*. 13.3, *p*=0.79) and ectopic pregnancies rate, % (2.6 *vs*. 2.1, *p*=0.98).

**Conclusion:** There was no difference in the results from oocyte donors and recipients. It is well known that this kind of program might be the way donors can access IVF, and for recipients may be the only option for pregnancy. A shared oocyte donation program that offers good and comparable results is fair and worth being stimulated.

**P-28. Quality of trophectoderme as predictive value in euploidy**

**Andrea Mesquita Lima** ^**1**^ **, Gleicyane Sousa Santos** ^**1**^ **, Sebastião Evangelista Torquato Filho** ^**1**^ **, Fábio Eugênio Magalhães Rodrigues** ^**1**^ **, Marcus Aurélio Bessa Paiva** ^**1**^ **, Tulius Augustus Ferreira de Freitas** ^**1**^ **, Eduardo Gomes Sá** ^**1**^

^*1*^ *BIOS - Centro de Medicina Reprodutiva do Ceará.*

**Objective:** Aneuploidy is a type of chromosomal anomaly that alters the normal amount of chromosomes present in an individual, representing one of the main causes of embryo implantation failure and miscarriage. In the process of in vitro fertilization (IVF), preimplantation genetic diagnosis (PGS) allows the selection of a genetically normal embryo for transfer. The aim of this study was to evaluate the quality of trophectoderme cells, according to their characteristics, such as: number of cells, compaction and regularity, and correlate with PGS results, analyzing whether the quality of trophectoderm cells can predict the formation of an euploid blastocyst, evaluated by genetic diagnosis.

**Methods:** Retrospective study, which included 126 embryos of couples with indication of IVF with PGS, between January 2021 and July 2021. The embryos were divided into euploids and aneuploids, according to a preimplantation genetic diagnosis report. The quality of trophectoderm was divided between A, B and C, with A being the best quality, and C to lower quality.

**Results:** Of the 126 embryos analyzed, 59 presented euploidy in the PGS result, and 67 presented aneuploidy in the PGS result. Among the euploid embryos, 36.7% presented trophectoderm with quality A, 49.7% of type B and 13.6% of type C.

**Conclusion:** The analysis showed that the qualities A and B of trophectoderm of blastocyst embryos seem to be a predictive factor for the embryo to be euploid.

**P-29. Immunohistochemical profile of endometrial biopsies performed on women submitted to assisted reproductive treatments**

**Carlos Alberto Link** ^**1**^ **, Carolina Giordani Andreoli** ^**1**^ **, Andréia Forsin Moro Torre** ^**1**^ **, Rafaela Amaro Link** ^**2**^

^*1*^ *Clínica ProSer Fertilidade e Reprodução Assistida.*

^*2*^ *Universidade de Passo Fundo.*

**Objective:** to describe the prevalence of abnormal findings on the immunohistochemical markers CD56 (present in natural killer cells), CD138 (chronic endometritis) and BCL6 (endometriosis) in the endometrium of infertile women with implantation failure or miscarriage, or even women that requested the test (controls).

**Methods:** cross-sectional study from endometrial biopsies performed form August 2020 through August 2021 on “Clínica ProSer Centro de Reprodução Assistida”, Porto Alegre/RS. Upon consent, ultrasonographic scan was performed to determine the best time to collect the biopsy, during the midluteal phase of the menstrual cycle. On natural cycles, urinary human chorionic hormone 5.000UI was given when a follicle of at least 1.7cm was seen, and biopsies were performed on day 5 of progesterone. On prepared cycles with 6mg of estradiol daily, micronized progesterone 600mcg daily was added after achieving endometrium thickness of at least 0.65cm, and biopsies were performed on day 5 of progesterone. The procedure was performed without the need for anesthesia, under ultrasonographic control, introducing a soft Wallace catheter through the cervix with syringe aspiration of small endometrial fragments. All Immunohistochemistry tests were performed on either “Medicina Digital Laboratório de Patologia” or “Instituto de Patologia”, both experienced pathology laboratories in Porto Alegre/RS. The markers analyzed were CD56, CD138 and BCL6. The results for CD56 were expressed in percentage, and all results equal or higher than 10% were considered positive/abnormal. The results for CD138 were expressed in positive/abnormal when at least one plasma cell was detected, or negative/absent. The results for BCL6 were expressed in H-Score, and considered positive/abnormal when H-Score was equal or higher than 1.4.

**Results:** between August 2020 and August 2021, 133 biopsies were performed, and available results for CD56, CD138 and BCL6 were 133, 133 and 100, respectively. In general, average age was 38 (from 27 to 48) and average body mass index was 24.3. While describing the indication for treatment, among the total of 133 patients, 59 had tubal factor, 37 had masculine factor, 25 had endometriosis factor, 28 had ovarian factor, meanwhile some patients had multiple factors. The prevalence of positive/abnormal CD56 results was 50% (66 out of 133 available tests). The prevalence of positive/abnormal CD138 results was 25% (33 out of 133 available tests). The prevalence of positive/abnormal BCL6 results was 24% (24 out of 100 available tests). We later divided the patients in three groups: at least one miscarriage (30 patients), at least implantation failure (85 patients), and no failure or miscarriage (control group, 18 patients). In the miscarriage group, the prevalence for abnormal CD56, CD138 and BCL6 was 30%, 30% and 32%, respectively. In the implantation failure group, the prevalence for CD56, CD138 and BCL6 was 50.5%, 24.7% and 23%, respectively. In the control group, the prevalence for CD56, CD138 and BCL6 was 50%, 16.6% and 12.5%, respectively.

**Conclusion:** the prevalence of abnormal findings on the immunohistochemical markers was remarkable, mainly CD56 (natural killer cells), when compared to the literature. There is no consensus among studies to determine the abnormal percentage for CD56 (natural killer cells), varying from 5 to 15%, and we used a 10% parameter. Most abnormal results were observed in the implantation failure group for all the three markers, pointing out the importance of endometrial evaluation before treating these group of patients. A larger study, including more patients in each group, could be planned in order to do statistical analyses and possibly demonstrate different prevalence between implantation failure, miscarriage and control groups.

**P-30. Association of overweight and obesity in seminal parameters**

**Tiago Cesar Mierzwa** ^**1**^ **, Debora Scaraboto** ^**1**^ **, Kahisa Natiele Fontana Dal Toso** ^**1**^ **, Lidio Jair Ribas Centa** ^**1**^

^*1*^ *Andrology Department of Fertility Clinic - Androlab; Curitiba - PR.*

**Objective:** Obesity is a global health problem and a possible worsening factor in male fertility due to the impact on the hormonal axis, metabolic alterations and testicular steroidogenesis. The objective of this study is to investigate the association between overweight and obesity in the seminal parameters of 1388 patients who sought a fertility clinic to perform a sperm analysis.

**Methods:** Retrospective study based on the analysis of surveys answered by patients who underwent sperm analysis at a private fertility clinic in Curitiba between November 2017 and June 2021. BMI was calculated using data provided in the survey thought the formula “BMI=weight(kg)/ height(m)²”. Patients whose BMI were higher than or equal to 18.5 and lower than 25.0 being considered as having adequate weight; those which BMI was higher than or equal to 25 and lower than 30 being considered overweight and those with a BMI higher than or equal to 30 were considered obese. This classification follows the recommendation of the Ministry of Health-Brazil. To facilitate the visualization of the results, patients were divided into two groups: those with BMI greater than 25 here called “high BMI” (n=965) and those with BMI below 24.9 called “Appropriate BMI” (n=423). The values considered normal were: seminal volume ≥ 1.5 mL, concentration ≥ 15 million sperm per mL, motility ≥ 32% of progressive motile sperm and morphology of at least 4% of normal sperm (Kruger's strict standard). Patients with varicocele, vasectomy or vasectomy reversal, patients who have or have had cancer, and those whose BMI was considered low (below 18.5) were not included in the study. Statistical analysis was calculated using the two-tailed chi-square test with a significance level of 0.05.

**Results:** 69.5% of patients were included in the “High BMI” group and 30.5% included in the “Appropriate BMI” group. Of all the overweight or obese patients, 7% had changes in ejaculate volume (n=72), 19% had oligozoospermia (n=182), 3% azoospermia (n=25), 37% asthenozoospermia (n=358), 49% teratozoospermia (n=476). Over the patients with good BMI, 5% had changes in ejaculate volume (n=20), 15% had oligozoospermia (n=65), 2% azoospermia (n=10), 38% asthenozoospermia (n=162) and 49% teratozoospermia (n=208).

**Conclusion:** There was no statistical difference between the two groups for any of the analyzed parameters. Patients in the “Appropriate BMI” group may have high rates of changes in the seminal parameters evaluated because most of these patients are infertile and, for this reason, they seek a specialized clinic to perform the spermogram. Thus, it cannot be proved that there is no association between overweight and obesity with seminal parameters. For further research, the objective is to select only patients who are not investigating infertility and/or who have never done another sperm analysis before, thus trying to minimize this bias.

**P-31. The use of progestins to inhibit spontaneous ovulation during ovarian stimulation: an alternative to a friendly protocol**

**Barbara Neudine Sandrini Baptista** ^**1**^ **, Vitória Luiza Batalhoti Brogiato** ^**1**^ **, Gabriel Monteiro Pinheiro** ^**1**^

^*1*^ *Universidade de Santo Amaro.*

^*2*^ *Disciplina de Ginecologia e Obstetrícia da Universidade de Santo Amaro.*

**Objective:** The aim of this work is to explain the differences between progestins and gonadotropin-releasing hormone (GnRh) blockers in stopping ovulation during a cycle of controlled hyperstimulation.

**Methods:** A bibliographic review of articles published since 2011 was carried out, related to databases such as Pubmed and Scientific Electronic Library Online (Scielo); including master's and doctoral tests. The research prioritized randomized or non-randomized clinical trials and systematic reviews; besides being limited to Portuguese, English and Spanish. The search in the databases was performed using the descriptors: female infertility; ovulation induction; progestins; progesterone receptors and reproduction, registered in the Health Sciences Descriptors (DECS).

**Results:** After analysing the characteristics of each of the 10 articles studied on the topic of interest, the efficiency of exogenous progesterone for blocking the peak of luteinizing hormone (LH) is remarkable, without affecting the number of oocytes collected or the quality of embryos obtained during ovarian stimulation. This is because progesterone is one of the main regulatory factors for the gonadotropin peak; therefore, its removal induces an acceleration of the LH pulse generator, while its administration delays its frequency. However, the use of progestins makes it impossible to transfer fresh embryos due to a serious impairment of endometrial receptivity; it is essential to associate the use of progesterone with the freeze-all protocol. This fact is evidenced in the studies analysed, such as the study by Begueria et al (2019). This was a prospective randomized clinical trial with 171 patients, 86 women constituting the study group and 87 the control group. The study group used medroxyprogesterone acetate (MPA) as an alternative to progesterone, which was administered in doses of 10 mg per day, while the control group administered GnRh antagonists for the pituitary block. Thus, the results obtained in the study group were 15.1±8.3 oocytes with a live birth rate of 22%, while the control group had 14.6 ± 7 oocytes with a live birth rate of 31%. No significant differences were found in the numbers of acquired oocytes and live birth rates in the study group compared to the control group. Thus, the inhibition of the LH surge with exogenous progesterone associated with the effectiveness of the so-called freeze-all protocol clearly indicates that progestins can be used to suppress the LH surge during ovarian stimulation in in vitro fertilization or in intracytoplasmic sperm injection, being a good alternative to GnRh agonists and antagonists, since these pituitary blockers have disadvantages, such as high cost and poor manageability, as the drug needs to be accurately prepared and administered only subcutaneously.

**Conclusion:** It is concluded that the administration of exogenous progesterone is effective, safe and economical to avoid extemporaneous LH surges during ovarian stimulation; however, it is mandatory to completely freeze the oocytes or embryos obtained and opt for delayed embryo transfer, to avoid the potential harmful effect of the hormonal environment on endometrial receptivity. Therefore, GnRh antagonists can be replaced by progestins, resulting in a low cost of treatment without significant changes in the success rate of live births.

**P-32. High sperm DNA fragmentation index is related to paternal age and may interfere with good-quality blastocyst formation rate**

**Luiza Mezzomo Donatti** ^**1**^ **, Norma Pagnoncelli Oliveira** ^**1**^ **, Nilo Frantz** ^**1**^

^*1*^ *Nilo Frantz Medicina Reprodutiva.*

**Objective:** High sperm DNA fragmentation index (DFI) is associated with several factors, including lifestyle and eating habits. DFI can be a male infertility cause, as the literature data suggest that sperm DNA damage reduces the chances of natural conception, especially with DFI higher than 25%. Therefore, the present study aims to evaluate the sperm DFI in different aged men and relate it to laboratory outcomes after assisted reproduction treatments.

**Methods:** Retrospective cohort study including 367 patients who underwent seminal collection at a human reproduction center between 2016 and 2021 and had the sample tested for sperm chromatin structure assay (SCSA) to evaluate sperm DFI. The DFI analysis was carried out through flow cytometry using acridine-orange probe. Men enrolled in this study were split in three different groups, according to age: <35 years old, 35-45 years old, and ≥45 years old (n= 63, 227 and 77 participants, respectively). The participants did not perform any therapy to reduce or prevent high sperm DFI. The mean DFI (%) among these groups was analyzed and compared. From the initial 367 patients, 255 were submitted to in vitro fertilization (IVF) cycles by intracytoplasmic sperm injection (ICSI) after DFI evaluation. For the patients submitted to IVF cycles, some seminal parameters (concentration, motility and volume), and fertilization rate, mean oocyte age and good-quality blastocyst formation rate (Bl1 or Bl2) were also analyzed. Blastocysts Bl1 are those classified as AA, AB or BA for inner cell mass and trophectoderm, respectively, and blastocysts Bl2 are those classified as BB or CB. One-way ANOVA was applied to compare the groups and p value ≤ 0.05 was considered statistically significant.

**Results:** Paternal age group ≥ 45 years old showed a significant higher sperm DFI when compared to group < 35 years old (28.6% *vs*. 20.8% *p*= 0.0135). Mean value for sperm DFI for the group 35-45 years old was 26.2% and did not differ significantly from <35 years old group (*p*= 0.1230) or from the ≥ 45 years old group (*p*= 0.4394). Despite not presenting a significant difference (*p*= 0.5987) the sperm DFI level trends to be inversely related with Bl1 or Bl2 formation on IVF cycles, since the oocyte age was similar among the studied groups (32.0 x 33.6 x 32.3 years old, respectively, *p*= 0.1183). Seminal concentration, motility and volume were evaluated and did not show significant difference among < 35 years old, 35-45 years old, and ≥ 45 years old groups (*p*= 0.6926, *p*= 0.8156 e *p*= 0.1153, respectively). Moreover, fertilization rate was not affected by sperm DFI level (69.3% *vs.* 74.6% *vs.* 76.1% *p*= 0.2108).

**Conclusion:** Advanced maternal age is already well established as an important conjugal infertility factor. Nevertheless, the advanced paternal age effects on male fertility are still controversial or remain undefined. This study shows a rise in sperm DFI level at advanced paternal age, although other seminal parameters are not modified. Men with sperm DFI higher than 25% can present some degree of infertility or natural conception difficulty due to high sperm chromatin damage. Therefore, paternal age should also be considered as a limiting factor when dealing with IVF outcomes.

**P-33. Morphokinetic parameters comparison between embryos from couples with high or low sperm DNA fragmentation index: what is the human eye missing during conventional morphological assessment?**

**Daniela Paes de Almeida Ferreira Braga** ^**1,2**^ **, Rodrigo Rosa Provenza** ^**1**^ **, Livia Silva Vingris** ^**1**^ **, Patricia Guilherme** ^**1**^ **, Assumpto Iaconelli Jr.** ^**1,2**^ **, Edson Borges** ^**Jr.1,2**^

^*1*^ *Fertility Medical Group. São Paulo/SP.*

^*2*^ *Instituto Sapientiae - Centro de Estudos e Pesquisa em Reprodução Assistida. São Paulo/SP.*

**Objective:** As paternal genome activation occurs late in the embryo, the negative impact of sperm DNA fragmentation on embryo development is more often observed in the outcomes of pregnancy, than in the potential for embryonic development. Several studies have demonstrated that embryo development is affected in subtle ways by a multitude of factors such as female and male partner ages, length of ovarian stimulation, embryo culture media and so on, apart from intrinsic embryonic quality itself. The introduction of new technologies into the in vitro fertilization (IVF) laboratory, such as the time-lapse imaging (TLI), has demonstrated to improve embryo culture condition and embryo development. With the development of TLI technology, and the possibility of assessing complete embryonic development, it has been hypothesized that sperm factors related to DNA fragmentation interfere with the speed and pattern of cell divisions, leading to slower embryos; something that would not be detected by conventional morphological embryo assessment. Despite many studies have addressed the relationship between SDF and IVF outcomes, little is known about the impact of SDF on embryo morphokinetics. The objective of this study was to investigate study whether TLI can identify morphokinetic events impacted by high sperm DNA fragmentation index (DFI).

**Methods:** This historical cohort study was performed in a private university-affiliated IVF center and included 978 zygotes cultured until day five in a TLS incubator between March/2019 and August/2020, derived from 118 patients undergoing ICSI as a result of idiopathic male factor infertility. Kinetic markers from the point of insemination were recorded. Generalized linear mixed models adjusted for potential confounders, followed by Bonferroni post hoc were used to compare timing of specific events in patients with low (<30%) or high (≥30%) sperm DFI. Recorded kinetic markers were: timing to pronuclei appearance and fading (tPNa and tPNf), timing to two (t2), three (t3), four (t4), five (t5), six (t6), seven (t7), and eight cells (t8), and timing to start of blastulation (tSB) and to blastulation (tB).

**Results:** Embryos derived from sperm samples with ≥30% DFI showed significantly slower divisions compared to those from <30% DFI (mean differences of 0.7h in tPNa, 1.2h in tPNf, 1.5h in t2, 2.5h in t3, 1.8h in t4, 3.3h in t5, 3.1h in t6, 3.2h in t7, 2.7h in t8, 8.4h in tSB and 3.8h in tB). The incidences of reverse or direct cleavages (9.3% *vs*. 4.4%, *p*=0.004, OR: 2.24, CI: 1.32 - 3.77) and multinucleation at 2-cell (18.9% *vs*. 12.0%, *p*=0.017, OR: 1.70, CI: 1.12 - 2.58) and 4-cell stages (14.2% *vs*. 6.4%, *p*<0.001, OR: 2.42, CI: 1.57 - 3.74) were significantly higher in embryos deriving from ≥30% DFI as compared to <30% DFI, respectively. The KIDScore ranked significantly different between embryos derived from samples with <30% or ≥30% DFI. Continuous DFI was positively correlated with all timings of specific events and with the incidences of abnormal cleavage patterns (OR: 1.042, CI: 1.025 - 1.059) and multinucleation at 2-cell stage (OR: 1.053, CI: 1.030 - 1.076), and inversely correlated with the KIDScore rank (B: -0.218, CI: -0.044 - -0.007). No significant differences were observed in clinical outcomes between the groups.

**Conclusion:** Embryo morphokinetic parameters are negatively impacted by high sperm DFI, resulting in delayed cell cleavage and blastulation. It is possible that the deselection of those embryos might have led to similar clinical outcomes between the groups, nevertheless further studies are necessary to confirm this hypothesis.

**P-34. Time of Morulation correlated with euploidy rate**

**Andrea Mesquita Lima** ^**1**^ **, Gleicyane Sousa dos Santos** ^**1**^ **, Sebastião Evangelista Torquato Filho** ^**1**^ **, Eduardo Gomes Sá** ^**1**^

^*1*^ *BIOS - Centro de Medicina Reprodutiva do Ceará.*

**Objective:** About 20 hours after intracytoplasmic sperm injection (ICSI), it is possible to verify the occurrence or not of fertilization, which is characterized by the presence of two pronuclei, one male and one female, which form the zygote. After this merger, the successive cleavages begin. Around the fourth day of in vitro cultivation, the embryo has about 12 to 30 cells, which are compact to each other. This compaction phenomenon, which characterizes the morbid stage, appears to be mediated by glycoproteins, and occurs around 96 hours after ICSI.The objective of this work is to evaluate whether the time of formation of the morula has any correlation with euploidia rate.

**Methods:** Retrospective study conducted from January 2020 to July 2021. A total of 252 morulas were analyzed, which evolved to the blastocyst stage, and were biopsied, with cell fragments sent for genetic analysis. All embryos analyzed in this study were in a time lapse incubator, in continuous culture medium. The morulas were separated into two groups according to the time of start of the morulation (tM): Group 1 (tM <90 hours) and Group 2 (tM > 90 hours). The results of the genetic analysis were inserted in each group to evaluate whether there was a correlation between the time of morulation with euploidy rate.

**Results:** Of the 252 mobiles evaluated, 146 showed time of morulation < 90 hours (Group 1) and 106 showed time of morulation > 90 hours (Group 2). Samples from Group 1 (tM <90 hours) had a 57.93% euplody rate, according to the result of the genetic analysis. Samples from Group 2 (tM >90 hours) showed 42.06%, according to the results of the genetic analysis.

**Conclusions:** The euploidy rate was higher in embryos that reached the morula stage before 90 hours of culture. This fact leads us to believe that morulation time seems to be a predictive factor for euploidy.

**P-35. Antinuclear antibodies in patients with endometriosis: a cross-sectional study in 94 patients**

**Laura Vilas Boas** ^**1**^ **, Carlos Bezerra Sobrinho** ^**1**^ **, Danilo Rahal** ^**2,3**^ **, Cesar Augusto Capellari** ^**3**^ **, Thelma Skare** ^**1**^ **, Renato Nisihara** ^**1,3**^

^*1*^ *Mackenzie Evangelical School of Medicine Paraná, Curitiba, Brazil.*

^*2*^ *Centro de Endometriose e Fertilidade Promater, Umuarama, Brazil.*

^*3*^ *Post Graduate Program in Gynecology and Obstetrics, Federal University of Paraná, Curitiba, Brazil.*

**Objective:** Markers of autoimmunity such as presence of autoantibodies have been found in patients with endometriosis. Among them are the antinuclear antibodies (ANA). Aim: To study a prevalence of ANA in a sample of endometriosis patients and its possible clinical associations.

**Methods:** Ninety-four endometriosis patients and 91 controls were studied for ANA, ENA profile (anti-Ro, anti-La, anti-Sm, anti -RNP) and anti-dsDNA. Epidemiological, clinical and staging data in endometriosis were obtained. Patients with autoimmune disorders were excluded. 

**Results:** Endometriosis patients had a 21.2% prevalence of positive ANA vs 5.4% in controls (*p*=0.001). ENA profile and anti-dsDNA were all negative. Patients with positive ANA were more asymptomatic (*p*=0.03) and had less dysmenorrhea (45% vs 68%) than the ANA negative group. No associations with disease duration, patient’s age or endometriosis stage were found.

**Conclusions:** There is a high prevalence of positive ANA in endometriosis patients. The presence of this autoantibody may be linked to a milder clinical expression of the disease.

**P-36. The fertility preservation: a case report of clinical pregnancy following 13 years of oocyte cryopreservation**

**Ricardo Marques de Azambuja** ^**1**^ **, Mariangela Badalotti** ^**1**^ **, Lilian Akemi Hirakawa Okada** ^**1**^ **, Marta Ribeiro Hentschke** ^**1**^ **, Shana Wander Flach** ^**1**^ **, Alvaro Petracco** ^**1**^

^*1*^ *FERTILITAT - Centro de Medicina Reprodutiva.*

**Objective:** To report a case of an ongoing pregnancy from oocyte cryopreservation following the slow procedure technique 13 years ago.

**Methods:** Case report study, performed at an assisted reproductive clinic in southern Brazil that included one patient. Data were collected from electronic records from a prospective database. The study was approved by the ethics and research committee of PUCRS, Porto Alegre.

**Results:** Before the vitrification technique became routine in the Assisted Reproduction laboratories, some few clinics around the world offered the possibility of oocyte cryopreservation through the slow procedure technique. From 2000 to 2008, our Center, in partnership with Saint Barnabas Clinic (USA), offered this technique to patients that wished to cryopreserve their oocytes for many different reasons. Thus, in 2008, a patient 36 years old was forwarded to our clinic for oocyte cryopreservation to delay maternity. Following ovarian stimulation with recombinant FSH and human menopause gonadotrophin in protocol with GnRH agonist, nine oocytes were recovered, and six were matured. The oocytes were cryopreserved in a choline-based solution with cryoprotector, and frozen using the slow cryopreservation method. In 2021, the patient then, at 49 years old, returned to the clinic with the desire to thaw her oocytes to get pregnant. She had oligomenorrhea and a normal uterus in the ultrasound. Her partner was with 66 years old and semen with volume=0.1 ml, concentration=90x106 sperm/ml, and motility=20%. The endometrium was prepared with estradiol valerate 6 mg/day oral; when it reached 9 mm of thickness, vaginal progesterone 600 mg/day was started. At this moment, the oocytes were thawed using the slow procedure method. Four of the six oocytes thawed (66%) survived and were inseminated; three fertilized and started their development. The transfer of two embryos happened on the third day of development: one embryo was starting compaction, and the other had six cells and 15% fragmentation, suitable for the day of development. The third embryo stopped its development following six days in culture. Twelve days after the transfer, βHCG was 64 mIU/dl, three days later that was 215 mIU/dl, and at the 18ºday it reached 2.415 mIU/dl. Fifteen days later, the ultrasound showed a gestational sac with one embryo with fetal heart beats, compatible with six weeks of gestation. Three weeks later, another ultrasound was compatible with nine weeks of gestation. The pregnancy is in progress, without complications.

**Conclusions:** Although the vitrification procedure has shown to be a better cryopreservation technique when compared to slow freezing, the latter represented an important role when patients wanted to cryopreserve oocytes in early 2000. Even many years later, this technique has shown its efficacy, preserving the viability and quality of oocytes stored in nitrogen tanks, allowing the oocyte utilization with success. After literature review, this case seems to be the largest interval between oocyte cryopreservation and its use with pregnancy.

**P-37. Male couple parenthood and assisted reproductive technology: a case report**

**Adriana Arent** ^**1**^ **, Isadora Badalotti-Telöken** ^**1**^ **, Catarina H Petracco** ^**1**^ **, Debora Farinati** ^**1**^ **, Alvaro Petracco** ^**1**^ **, Mariangela Badalotti** ^**1**^

^*1*^ *Fertilitat - Medicine Reproductive Center - Porto Alegre - RS.*

**Objective:** To report a case of an ongoing pregnancy from oocyte cryopreservation following the slow procedure technique 13 years ago.

**Results:** A male couple looking for fertility treatment, one partner 37 years old and another partner with 34 years old. They are aware of the necessity of egg donation and surrogacy maternity. The couple has a medical and psychological evaluation in order to assess the couple's relationship to determine stability and their commitment to becoming parents through assisted reproduction. The couple used an egg-sharing program. The egg donor was a 30 years old patient who was either under IVF treatment. Once selected, the donor will undergo an intensive screening that includes medical, psychological, genetic, and infectious disease tests and also meet with an independent reproductive attorney. The ovarian stimulation was carried out with human menopausal gonadotrophin; 0,25 mg of ganirelix was used during six days, from day six. There were 28 follicles >16mm in diameter. When at least three follicles had a diameter greater than 17 mm, final oocyte maturation was triggered by administering 0.1 mg triptorelin, and follicular aspiration was performed 35 hours later by transvaginal ultrasound. Twenty-eight follicles were aspirated, 26 eggs were retrieved, and 24 were mature eggs (MII). The couple received 12 MII oocytes, and the egg donor kept the other 12 MII oocytes. Both partners had equal desires for biological fatherhood and chose to inseminate equal numbers of oocytes to transfer one embryo from each partner each time. They know their embryos could not be mixed to preserve the genetic identity of the child. Eight embryos reached a blastocyst stage and were frozen. For the surrogacy maternity, the couple had a friend who offered to carry the pregnancy. According to Brazilian legislation, surrogate mothers should be a relative, but none of them have one relative in their families. In these cases, an authorization of the state medical council is necessary, which was provided. The surrogate mother was a 47 years old woman who already had two adult sons. Her medical evaluation didn't show any contra-indication to carry a pregnancy. In addition to the medical evaluation, a psychological assessment was done to explore the couple's relationship with the gestational carrier, particularly whether they have a trusting and open relationship with her and what her future relationship (if any) will be with the children. The endometrial preparation of surrogate mother was run with increasing doses of estradiol valerate (beginning with 2mg and progressively reaching a daily dose of 8 mg), and when endometrium reached 10 mm of thickness, 600 mg of micronized progesterone per day was started, and six days after a single embryo in blastocyst stage was transferred, which result in a single pregnancy.

**Conclusion:** The desire to have biological children is not unique to the traditional nuclear family. The ongoing advancement and increasing availability of assisted reproductive technologies (ART) make it possible for male couples and single men to have biological children of their own, using many of the same cutting-edge techniques that have helped infertile heterosexual couples become parents for decades. Counseling these couples on ART's medical and emotional demands with a gestational carrier and oocyte donor is a vital component of pretreatment preparation.

**P-38. Obese women seeking ART: is there a higher treatment cost?**

**Victoria Campos Dornelles** ^**1**^ **, Isadora Badalotti-Teloken** ^**1**^ **, Marta Ribeiro Hentschke** ^**1**^ **, Natalia Fontoura de Vasconcelos** ^**1**^ **, Alvaro Petracco** ^**1**^ **, Mariangela Badalotti** ^**1**^

^*1*^ *Fertilitat - Centro de Medicina Reprodutiva.*

**Objective:** To evaluate the impact of female obesity on assisted reproduction techniques (ART) costs.

**Methods:** Retrospective cohort study performed at an assisted reproductive clinic in Brazil. Data were collected from electronic records from 2013 to 2020. A total of 2653 in vitro fertilization (IVF) cycles were included. Samples were divided into three groups according to female body mass index (BMI): Group 1, eutrophy (n= 1940), Group 2, overweight (n= 530), and Group 3, obesity (n= 183). Variables were compared between groups and were expressed as mean±SD or n(%). Student t-test, ANOVA, and Chi-square tests were applied. Statistical significance was defined as *p*<0.05.

**Results:** Comparing groups 1, 2, and 3, the following results were found, respectively: IVF canceled cycles (6.9% *vs*. 7.8% *vs*. 10.4%, *p*=0.001) was lower in eutrophic group (*p*=0.001), as the mean of total gonadotropin doses (1678 UI *vs*. 1779 UI *vs*. 1796 UI, *p*=0.000). Analyzing the average gonadotropin costs in Brazil, each UI of menotropin (hMG) is around R$ 1.84, and the average recombinant FSH (FSHr) and FSHr + LHr costs per UI are around R$ 2.49. Then, the average value of these three gonadotropins is R$ 2.27/UI. Thus, the gonadotropin costs were estimated and compared between groups: R$ 3.809,06 per eutrophic patient vs. R$ 4.038,33 per overweight patient *vs*. R$ 4.076,92 per obese patient (*p*=0.003).

**Conclusion:** The higher was the BMI, the higher was the cycle cancelation and the gonadotropin mean dose needed, and, hence, more expensive was the treatment, even with limited sample size. This study highlights the importance of also considering the higher treatment costs, beyond all the well-known risks regarding weight excess, fertility, and pregnancy, before starting IVF treatments.

**P-39. Evaluation of the ZyMōt** ^**®**^ **Multi ability to select sperm with low DNA fragmentation**

**Beatriz Müller Nunes Souza Souza** ^**1**^ **, Camila Pinho Pompeu** ^**1**^ **, Luiz Mauro Gomes de Oliveira** ^**1**^ **, Leticia Emi Kadomoto Ferreira** ^**1**^ **, José Fernando Macedo** ^**1**^

^*1*^ *Clínica Reproferty.*

**Objective:** This study aims to evaluate the ability of the Zymōt^®^ Multi device (850µL) to decrease the rate of sperm DNA fragmentation in patients who underwent Assisted Reproductive Technology (ART).

**Methods:** Patients (n=8) undergoing in vitro fertilization (IVF) treatment with a high rate of sperm DNA fragmentation (>20%) were selected. DNA fragmentation analyzes were performed before and after seminal processing with ZyMōt^®^ Multi device (850µL), regardless of sperm morphology and period of ejaculatory abstinence. Sperm DNA fragmentation test was performed by chromatin dispersion methodology with the Canfrag^®^ kit. After calculating the avarage of the fragmentation results of groups before and after ZyMōt^®^, statistical analysis was performed between them using Bioestat 5.0 software, through ANOVA and Tukey two-way test, using a value of *p*≤0.05. Inclusion criteria: presence of motile sperm and sperm concentration equal or above 0.1x106/mL. Exclusion criteria: samples with only immotile sperm or with a sperm concentration below 0.1x106/mL were excluded.

**Results:** Considering all analyzed patients, the highest rate of sperm DNA fragmentation observed before ZyMōt^®^ was 55% while the lowest was 21%. In after ZyMōt^®^ group, the highest percentage of fragmentation observed was 19% and the lowest was 7%. In the group before ZyMōt^®^ the avarage for sperm DNA fragmentation was 34%, while in the group after ZyMōt^®^ was 14%, reaching a significant reduction (*p*≤0.05) of 20% between evaluated groups.

**Conclusion:** The present study demonstrated that group after ZyMōt^®^ analysis resulted in a significant reduction in sperm DNA fragmentation. Though, the studies needed to evaluate a larger number of samples, also correlating with period of ejaculatory abstinence, as it is known to influence the increase in DNA damage. Therefore, based on the results obtained, the Zymōt^®^ Multi device (850 µL) proved to be effective in separating sperm with less DNA damage, and may be a beneficial seminal processing option for patients undergoing IVF treatment.

**P-40. Are there differences in Endometrial Microbiota Profile of Brazilian IVF patients compared to European patients?**

**Aline Rodrigues Lorenzon** ^**1**^ **, Marjorie Fasolin** ^**1**^ **, Selma de Fatima Moreira Tsuji** ^**1**^ **, Leticia Silva** ^**1**^ **, Mauricio Barbour Chehin** ^**1**^

^*1*^ *Huntington Medicina Reprodutiva - Eugin Group. São Paulo, SP, Brazil.*

**Objective:** The uterine microbiota is an ascending, although controversial, area of research. Individual/ethnicity characteristics, such as genital infections, dietary and physical exercises routine and sexual habits may have profound impact on endometrial microbiota. The aim of this study is to investigate if the endometrial microbiota profile from Brazilian IVF patients are similar from that referenced by an available commercial screening test for this purpose in Brazil.

**Methods:** This prospective, observational cohort study, included patients undergoing IVF cycles that performed the EMMA (Endometrial Microbiome Metagenomic Analysis, Igenomix^®^) test according to medical referral from July 2019 and July 2021 at Huntington Medicina Reprodutiva clinic, in São Paulo, Brazil. The microbiota profile were compared with the results published by Moreno and collaborators, 2016 (that analyzed n=41 endometrial fluid samples from 35 patients undergoing IVF in Europe). The results are represented as proportion of each bacterial type by sample (%) and mean±standard deviation. Fisher exact test was applied for statistical analysis.

**Results:** Four hundred twenty nine (429) samples, from 399 Brazilian patients were included in our cohort. Mean age was 39.19±4.65 year old. Normal microbiota was reported in 21.21% (91/429) of our tests, in contrast to 43.90% (18/38 samples from 35 IVF patients) from the study of Moreno, 2016. This result show a lower proportion of normal microbiota profile in Brazilian patients (*p*=0.04) according to the established threshold from EMMA test. Mean age of normal microbiota was 38.73±5.0 years old (similar to 40.06±3.47 yo from Moreno, 2016). Altered microbiota (dysbiosis and abnormal profile) was reported in 48.95% (210/429) of the tests, similar to 48.78% from Moreno’s study). Mean age for altered microbiota patients was 39.02±4.04 years old (similar to Moreno’s study 39.00±5.09). However, in our study 128/429 (29.84%) of the samples were reported as ultra-low biomass (n=126), not informative (n=01) or invalid (n=01) samples. There is no information of the detection of ultralow biomass samples in Moreno’s study, however they reported 3/41 samples did not amplify properly (7.3%). Ultra low-biomass are considered as low flora samples by the result interpretation’s section from EMMA test. Nevertheless, from the biological point of view, the absence of discernment of any bacterial genera may be interpreted as an inconclusive report. The proportion of inconclusive results was higher in our population (29.94% versus 7.3%, p=0.0015).The top 5 bacterial genera founded in the endometrial fluid from fertile patients (egg donors) in the study from Moreno, 2016 were: *Lactobacillus* (71.7%), *Gardnerella* (12.6%), *Streptococcus* (3.2%), *Bifidobacterium* (3.7%) and *Prevotella* (0.866%). The results from patients undergoing IVF in Europe were shown as *Lactobacillus* Dominant (≥90%) or non-dominant (<90%) only (without the description of others bacterial genera). In our IVF patients, the top 5 bacterial genera were also *Lactobacillus* (64.98%), *Gardnerella* (8.29%), *Streptococcus* (3.11%), *Bifidobacterium* (2.02%) and Klebsiella (1.94%), instead of *Prevotella*, as reported in egg donors patients in Moreno’s study.

**Conclusions:** Normal microbiota profile in Brazilian IVF patients is lower than in European patients, according to the thresholds established by Moreno and collaborators, 2016 (21.21% versus 43.90%). The proportion of ultra-low biomass/non-informative profile was higher in our population (29.84% versus 7.3%). In summary, the clinical relevance of endometrial microbiome analysis remains to be further investigated and caution is recommended when analyzing Brazilian patient’s reports. The implications of the lifestyle and the potential presence of different types of bacterial genera must be considered for result interpretation.

**P-41. What the psychologist is talking about. Documentary research in Assisted Reproduction**

**Patricia Marinho Gramacho** ^**1**^ **, Marina de Moraes e Prado Morabi** ^**2**^ **, Kátia Straube** ^**3**^

^*1*^ *Humana Medicina Reprodutiva.*

^*2*^ *PUC - GO.*

^*3*^ *Instituto Suassuna.*

**Objective:** This study aims to make a documentary analysis of psychological reports presented from October 2017 to January 2020, characterizing the emotional contents emerging in the speech of hetero-affective and homo-affective couples, in the process of donating or receiving eggs, during a specific psychological listening in an assisted reproduction clinic, computing 151 reports.

**Methods:** A guiding script was built from the basic questions necessary to investigate this process, which covered the following topics, within a semi-directed interview: affective history of the couple; generational family history; history of infertility and the consequent need to donate or receive eggs; in addition to the story of a child's desire. A proposal was made to start the interview for one of the topics, according to the couple's choice, to guide the characterization of emotional contents.

**Results:** The results showed that the most chosen topics to start the interview were: affective history of the couple, for same-sex donor couples; history of infertility, for hetero-affective couples; and affective history of the couple and history of infertility, for recipient couples, homo or hetero-affective.

**Conclusion:** The importance of this characterization lies in the possibility of contributing to improve the welcoming process in psychological care for these patients. The documentary analysis also shows that the decision to start a family is not just the decision of two people who intend to share their lives, but results from a system of families, as Dunker (2020). Maintains, from exchanges, alliances and obligations, from circulation and transmission of symbolic and material goods that make the arrival of children a critical moment in the conjugality-parenting transition.

**P-42. Serum concentrations of complement system c5a component in patients with endometriosis: Clinical-laboratory association**

**Danilo Rahal** ^**1**^ **, Fabiana Andrade** ^**2**^ **, Renato Nisihara** ^**2**^

^*1*^ *Centro de Endometriose e Fertilidade Promater, Umuarama, Brazil.*

^*2*^ *Clinical Hospital, Federal University of Paraná, Curitiba, Brazil.*

**Objective:** Endometriosis (EM) is a gynecological disorder that presente importante immune dysregulation in its pathophysiology. Recent studies indicate that Complement System may play a significant role in the immune process related to peritoneal clearance and inflammation in EM. C5a is a potent anaphylotoxin molecule of complement associated with the development of inflammatory disorders, however its possible impact on EM development requires further investigations.

**Methods:** A total of 94 patients with EM (stage I n=27, stage II n=24, stage III n=23 and stage IV n=20) and 50 healthy controls were assessed for C5a serum levels by ELISA assay. In addition, clinical and demographic data were also included in the analyses.

**Results:** C5a serum levels were higher in patients with EM than in controls (39.5 ng/mL vs. 26.0 ng/mL; *p*<0.0001), but not different between the EM stages. Also, no association was observed between C5a serum concentration with presence of symptoms, age, symptom time or infertility time.

**Conclusions:** our findings suggest a role for C5a in the pathophysiology of EM.

**P-43. Assisted human reproduction techniques for serodiscordant couples: Case report**

**Bruna Gomes dos Santos** ^**1**^ **, Welington Alberto Balla** ^**1**^ **, Pablo Henrique de Assis Santos** ^**1**^ **, Silvana dos Santos Meyrelles** ^**2**^

^*1*^ *Biofert Centro de Reprodução Humana.*

^*2*^ *Universidade Federal do Espírito Santo.*

**Objective:** The acquired immunodeficiency syndrome, worldwide known as AIDS is the most advanced infection stage due to the Human Immunodeficiency Virus (HIV). One of the major concerns of the serodiscordant couples, in which only one is HIV positive, is a possibility to have an unhealthy baby. Fortunately, with advance in the Assisted Reproductive Technology (ART), it is possible to provide a safe pregnancy to those serodiscordant couples. Objective: To report the use of an assisted reproductive technique in a HIV serodiscordant couple.

**Methods:** Seropositive man and seronegative woman, searched our fertilization service to obtain a safe first pregnancy. After consultation and orientations, the sperm collected by the man was first submitted to macroscopy analysis to evaluate volume, liquefaction time, pH, color, and viscosity and submitted to sperm concentration and motility microscopy evaluation. After seminal analysis, the sample was submitted to sperm-wash technique, that consisted in sample centrifugation with 1500 rpm for 20 minutes (Fanem centrifuge model 206-R) in concentration gradient (45% and 90%). After, the resultant sediment was washed with 1 ml of culture media, centrifuged with 1500 rpm for 10 minutes and resuspended in 0.5 ml of media. Then, the sperm motility and concentration were assessed post spermatic capacitation. At the end of procedure, a 0.3 ml sample was separated and sent to specialized laboratory to check DNA and RNA virus presence through PCR technique. After negative PCR result and the specialized laboratory permission to use the spermatozoids in the assisted reproductive procedure, in vitro fertilization technique started. Briefly, the wife was submitted to follicular aspiration, and 09 oocytes were retrieved. The oocytes were put in a culture plaque with appropriated media. To allow the classic in vitro fertilization, an aliquot of 1.2 ml from seminal sample was put with the oocytes in the same culture plaque. After spermatozoids and oocytes fuse together, pre-embryo developed for a 3-day period and five embryos were vitrified to ensure their preservation. After 2 cycles, two embryos were devitrified and transferred to a woman uterus.

**Result:** After thirty-eight weeks and 6 days of gestation, the woman gave birth a health and HIV seronegative child.

**Conclusion:** This case report showed the extreme importance of Assisted Reproduction Technology to provide the necessary safe to avoid baby HIV contamination in serodiscordant couples that wish to become a parent.

**P-44. A qualitative investigation of the patient’s expericence undergoing to IVF/ICSI cycles with donated oocytes**

**Fernanda Kunrath Robin** ^**1**^ **, Sandra Maria Cezar Leal** ^**2**^ **, Nilo Frantz** ^**1**^ **, Gabriella Mamede Andrade** ^**1**^

^*1*^ *Nilo Frantz Medicina Reprodutiva.*

^*2*^ *Universidade do Vale do Rio dos Sinos.*

**Objective:** Oocyte donation is one of the last resorts in IVF treatment for couples challenged with infertility problems. Postponement of motherhood, genetic and oncological diseases and early ovarian failure have been the main reasons for the increase number of IVF cycles with donated oocytes. The objective of the current study was to investigate how heterosexual couples or women faced the ovodonation process.

**Methods:** A qualitative descriptive study was carried from January 2017 to February 2020 in five units of a reproductive center with the head office in Porto Alegre, RS, Brazil. All couples who underwent ovodonation were invited to join the research but, those who were not successful with the treatment and who did not reply to the research invitation were excluded. During the study period, 222 ovodonation cycles were carried out, and of these, 151 women became pregnant, but 42 women were excluded due to pregnancy loss or because it was not possible to establish contact, remaining in the study 109 women. From this final number, 21 women were randomly chosen, one of whom had no partner, totaling 41 participants. Initial phone contact was made with the 21 women talking about the research, only one woman did not accept to participate, thus, she and her husband were excluded, therefore, 39 questionnaires and Informed Consent Form were sent. A total of 22 responses were obtained: 16 women and six men. The outcomes were analyzed using Iramuteq software. Six main topics stood out: (1) Challenges faced by couples undergoing treatment; (2) Dream of motherhood being possible with donated eggs; (3) Means of acquiring information; (4) The course during the ovodonation treatment; (5) Positive result contributes to overcoming difficulties; (6) Tell or not to tell the child about the conception with a donated egg.

**Results:** Through the results can be seen that there are many challenges faced by couples who are undergoing ovodonation treatment. Initially, the decision making, which in some cases, comes after long periods of IVF cycles with their own eggs that have ended in failure and frustration. As a result, the initial difficulty of accepting to generate a child without the woman's owns material, experiencing a period with contradictory feelings, the relief and happiness of having the possibility of forming a family, accompanied by shame, frustration and the difficulty of accepting the abandonment of the load genetics. The waiting time for the compatible phenotype was cited as a factor that generates high expectations and anxiety. The experience of pregnancy for these couples was a period of great joy, overcoming the difficulties experienced so far. The couples surveyed reported little information on the topic, highlighting the need to have places where it was possible to find reliable and up-to-date information, as well as testimonials from people who have already undergone treatment, considering that these aspects would make the process easier to be faced. Regarding the revealing to the child about ovodonation, the answers were well divided between not telling, telling and that they have not yet thought about the subject since not all babies were born or are still very small, this topic was very little commented on by the respondents, appearing to cause stress and anxiety.

**Conclusions:** Based on the research results it was possible to understand that the ovodonation treatment generated different emotions and ethical considerations in each step experienced. Themes generated from this research data analysis corroborate with researchers experience and reinforce the need for further studies with patients who have already experienced this process.

**P-45. Is there any harm of injecting a maximum of eight oocytes in an ICSI cycle?**

**Pedro Augusto Araújo Monteleone** ^**1,2**^ **, Alecsandra Prado Gomes** ^**1**^ **, Mariana Gomes Fujii** ^**1**^ **, Janaina Gisela de Brito Cunha** ^**1**^ **, Hamilton de Martin** ^**1,2**^ **, Carlos Augusto Zarate Nissel** ^**1,2**^ **, Mayra Satiko Lemos Nakano** ^**1,2**^ **, Tatiana Carvalho de Souza Bonetti** ^**1,3**^

^*1*^ *Centro de Reprodução Humana Monteleone, São Paulo - SP.*

^*2*^ *Departamento de Obstetricia e Ginecologia da Faculdade de Medicina da Universidade de São Paulo (FMUSP), São Paulo - SP.*

^*3*^ *Departamento de Ginecologia da Universidade Federal de São Paulo, Escola Paulista de Medicina (UNIFESP-EPM), São Paulo - SP.*

**Objective:** A recent update on ethical rules published by the Federal Council of Medicine (CFM) for the use of assisted reproduction techniques (ART) in Brazil (RESOLUÇÃO CFM Nº2.294, 27/05/2021) determines that the total number of embryos generated in an in vitro fertilization (IVF) cycle must do not exceed eight. To guarantee that, a maximum of eight MII-oocytes must be injected. This study aimed to evaluate clinical outcomes of IVF cycles according to the number of MII-oocytes injected, women's age and severe male factor.

**Methods:** This retrospective study reviewed data from ICSI cycles from May 2018 to July 2021. It included ICSI cycles with own or donated fresh MII-oocytes. Poor ovarian response (less than five MII-oocytes injected) or embryo biopsy for PGT-A cycles were excluded. Embryos were cultured in the time-lapse system incubator (Embryoscope^®^) and fresh and/or frozen-thawed embryos transfers were performed. A total of 1,221 ICSI cycles included were firstly divided into two groups according with women's age: the first group were composed by young women (<38 years old, mean of 34.3±3.0) and the second group included advanced maternal age (AMA) (≥38 years old, mean of 40.0±1.9). For donated oocyte cycles, the donor age was considered. Then, cycles were also divided based on number of MII-oocytes injected as standard-responders (5-8 MII oocytes inseminated) or high-responders (≥9 MII oocytes inseminated) and the presence, or not, of severe male factor (SMF), defined as sperm concentration <2 million/mL. Finally, cycles were classified into eight groups: G1 (young, no-SMF and standard-responder, n=185), G2 (young, no-SMF and high-responder, n=241), G3 (young, yes-SMF and standard-responder, n=18), G4 (young, yes-SMF and high-responder, n=28), G5 (AMA, no-SMF, standard-responder, n=158), G6 (AMA, no-SMF, high-responder, n=106), G7 (AMA, yes-SMF, standard-response, n=11) and G8 (AMA, yes-SMF, high-responder, n=2). Due to the small number of cycles available for G7 and G8, data were described but it were not statistically analyzed. The ongoing pregnancy rates (OPR) considered all fresh and/or frozen-thawed embryo-transfers (ET) realized for each stimulation cycle.

**Results:** The mean number of MII-oocytes injected in standard-responders were: G1: 6.7±1.1, G3: 6.3±1.3, G5: 6.3±1.1 and G7: 6.5±1.0; and in high-responders were: G2: 13.9±5.4, G4: 17.6±9.9, G6: 13.2±5.1 and G8: 16.0±8.4. The blastocyst development rate per MII-oocyte injected was 43.0% and it did not differ among groups (*p*=0.300), as the mean number of embryos transferred per cycle (1.9 to 2.4, mean of 2.1±1.1, *p*=0.064). The OPR per started cycle were higher in younger women (G1: 48.6%, G2: 56.4%, G3: 38.9%, G4: 64.3%) than AMA (G5: 18.4%, G6: 32.1%, G7: 18.2% and G8: 50.0%) as expected. The mean number of surplus embryos cryopreserved in successful cycles (G1: 2.5±1.3; G2: 5.3±3.6; G3: 2.6±0.5; G4: 7.3±6.3; G5: 2.4±1.4; G6: 5.8±3.2) are significantly lower in standard-responders compared to high-responder's cycles (*p*<0.001). However, the percentage of cycles with surplus embryos cryopreserved (G1: 68.9%; G2: 77.2%; G3: 71.4%; G4: 83.3%; G5: 55.2%; G6: 79.4%) were high in all groups.

**Discussion:** Our findings have shown that the blastocyst development rates are similar in all groups, suggesting that the potential for blastocyst development does not depend of the number of MII-oocytes collected or women's age, even if SMF is present (the group with AMA and SMF was not evaluated due to small number of cycles). In our casuistic, we also found that all groups had surplus embryos cryopreserved in the majority of successful cycles, even if the mean number of cryopreserved embryos are smaller for the standard-responders compared to high-responders. Our findings suggest that injecting a maximum of 8 MII-oocytes could reduce the mean number of embryos cryopreserved but does not achieve the goal of reducing cycles with surplus embryos cryopreserved.

**P-46. Epidemiological profile of infertile patients treated at a public tertiary care hospital**

**Gabriela de Oliveira Ribeiro Lima** ^**1**^ **, Isa Alves Rocha** ^**1**^ **, Karina de Sá Adami Gonçalves Brandão** ^**1**^ **, Renata Lopes Britto** ^**1**^ **, Hortensia Souza Guedes de Oliveira** ^**2**^ **, Fernanda Veiga Rodeiro Nery** ^**2**^

^*1*^ *Hospital Universitário Professor Edgard Santos - Universidade Federal da Bahia.*

^*2*^ *Escola Bahiana de Medicina e Saúde Pública.*

**Objective:** In the first half of the 21st century, about 50 million couples worldwide live in the context of infertility. The inability to conceive and, consequently, the inability to plan a family has a negative impact on individuals, their social and emotional context and interpersonal relationships, and can bring about severe conflicts and, sometimes, ruptures within the family circle. In this scenario, it is very important to remember the difficulties that infertile couples face during their journey in search of diagnosis and treatment, contributing to the psycho-emotional stress to which these couples are subjected and it is valuable to know the profile of patients who seek care for infertility. The study consists of evaluating the epidemiological profile of infertile patients followed up in a public and university service and is categorized as a descriptive retrospective cross-sectional observational study. Objectives: To describe the epidemiological profile of women followed up in an infertility outpatient clinic and estimate the sociodemographic and biological factors associated with pregnancy failure.

**Methods:** This is a descriptive retrospective, cross-sectional observational study, with evaluation of secondary data contained in the medical records and their respective records of the infertility outpatient clinic in the period between March 2018 and March 2020. Data were collected at from a personalized data collection instrument, which addressed the social, demographic and clinical factors of the monitored patients and some parameters of their partners, as well as the diagnoses concerning the disease in question (infertility).

**Results:** The sample consisted of 123 medical records, in which the mean age found in the patients was 33.4 years (SD±5.4). About 64.2% of women reported regular menstrual cycles and 56% of them were nulliparous. It was found that most patients in the study had ovulatory cause as a factor associated with infertility, making up 50.8% of the sample. And after the treatments, 10% of the total sample evolved with pregnancy.

**Conclusions:** The study concluded that among the patients seen at the outpatient clinic, most were young, with a mean age of 33 years, nulliparous, with ovulatory factor as the main cause of infertility.

**P-47. Reasons for sperm cryopreservation in a private semen bank**

**Tiago Cesar Mierzwa** ^**1**^ **, Débora Scaraboto** ^**1**^ **, Isadora Terumi Saruhashi** ^**1**^ **, Ana Caroline Possobon** ^**1**^ **, Lidio Jair Ribas Centa** ^**1**^

^*1*^ *Andrology Department of Fertility Clinic - Androlab - Curitiba - PR.*

**Objective:** Sperm cryopreservation is a procedure indicated for patients who will undergo gonadotoxic treatments and who wish to preserve their fertility. The aim of this study was to investigate the reasons that led patients to cryopreserve sperm in a private sperm bank in Curitiba for the last 40 years (between 1980 and 2020).

**Methods:** Retrospective study based on clinical data of patients who cryopreserved sperm in a private sperm bank in Curitiba between 1980 and 2020. Data from medical records were analyzed searching the reason for cryopreservation.

**Results:** 1007 patients were evaluated between 1980 and 2020. The distribution among the reasons for sperm freezing was: 53.9% are oncologic patients (n=393), 14.8% are patients who froze their gametes before undergoing vasectomy (n=108), 13.3% cryopreserved before procedures of IVF, ICSI or IUI (n=97), either due to absence on the day of the procedure or difficulties on semen collection, 8% frozen surplus sperm after testicular or epididymal biopsies (n=58), 3.4% (n=25) froze to perform the post-washed viral load test (HIV or Hepatitis), 1.9% before undergoing varicocelectomy surgery (n=14), 1.4% after vasectomy reversal (n=10), 1, 4% for social causes (n=10), 0.8% hormonal pretreatment for transgender women (n=6), 0.5% for testicular injury (n=4) and 0.4% for mumps (n=3 ). Another 38% are included in unknown causes, not registered or not disclosed by the patient (n=279).

**Conclusions:** The survey of the data from the sperm bank of this private clinic showed that patients diagnosed with cancer or having unidentified tumors are the majority of those who seek for semen cryopreservation. This reinforces the need to disclose this kind of intervention between oncologists, in order to improve patients' access to fertility preservation conditions.

**P-48. Couples' weight excess and *in vitro* fertilization: is there an impact on clinical outcomes?**

**Victoria Campos Dornelles** ^**1**^ **, Isadora Badalotti-Teloken** ^**1**^ **, Marta Ribeiro Hentschke** ^**1**^ **, Natalia Fontoura de Vasconcelos** ^**1**^ **, Alvaro Petracco** ^**1**^ **, Claudio Teloken** ^**1**^ **, Mariangela Badalotti** ^**1**^

^*1*^ *Fertilitat - Centro de Medicina Reprodutiva.*

**Objective:** Provide theoretical framework about the drugs Clomiphene Citrate and Letrozole, comparing them in terms of effectiveness and live birth rate.

**Method:** Literature review, with search in BVS, MEDLINE and PUBMED databases, including cohort studies, case-control studies, randomized clinical trial, clinical practice guide, master's and doctoral theses and systematic review, between the years 2010 to 2020. Case reports were excluded.

**Results:** Infertility is a growing challenge in Brazil, due to the choice of women in getting pregnant later. Therefore, it is important to discuss the available therapeutic options. The manuals use Clomiphene Citrate (CC) as the first line of treatment for ovulation induction, but other countries have been using Letrozole exhaustingly, a drug prescribed as "off-label" in Brazil. CC has a weak estrogenic effect, stimulating ovulation by releasing GnRh and gonadotropins. Letrozole, on the other hand, is an aromatase inhibitor, used by CC-resistant women, which acts by increasing follicular sensitivity to FSH. In comparison, it was observed that the ovulation rate of letrozole and CC did not differ significantly. It was also observed that CC caused an extremely thin endometrial thickness in 50% of women. The rate of pregnancies that did not progress with the use of CC (18.5%) was higher compared to Letrozole (11.5%). In several clinical trials, women who received letrozole had higher live birth rates compared to CC.

**Conclusion:** Letrozole is as effective as CC, especially in ovulation and live birth rates, plus it demonstrated superiority in terms of endometrial thickness and gestational evolution. Hence, this drug should be considered a good option for ovulation induction.

**P-49. Birthweight and gestational age of singletons and multiple pregnancy newborns at CLINIFERT**

**Vanessa Nayane Perez** ^**1**^ **, Mila Harada Ribeiro Cerqueira** ^**1**^ **, Gustavo Cerqueira e Silva** ^**1**^ **, Ana Clara Esteves** ^**1**^ **, Vanessa Cristina Freitas Moreno** ^**1**^ **, Kazue Harada Ribeiro** ^**1**^

^*1*^ *Clinifert Centro de Reprodução Humana.*

**Objective:** The aim of this study was to compare the data of birthweight and gestational age of singleton and multiple pregnancy newborns in our center to those in literature.

**Methods:** Pregnancy was defined by the visualization of gestational sac in the first ultrasound. We took note of the number of embryo transfers that resulted in pregnancy, total number of embryos transferred per pregnancy, number of newborns and compared the data on newborns gestational age and birthweight of singletons and multiple pregnancy newborns at Clinifert between 2012 and 2019, from both fresh and frozen embryo transfers (FET). Once our focus is to compare newborn data from single and multiple pregnancies, regardless of the nature of embryo transfer, we did not considered it necessary to make the distinction between fresh and frozen cycles.

**Results:** The mean age in both groups was 34 years old. We had 446 singleton pregnancies resulting from the transfer of 1026 embryos, from which 110 resulted in abortion. That leaves us with 336 newborns: 24 from single embryo transfer (SET), 205 from double embryo transfer (DET), 98 from triple embryo transfer (TET) and 9 from quadruple embryo transfer (QET). Mean birthweight for singletons was 3112.29g±511.36, and mean gestational age was 38.57±1.45 weeks; 28 (8.4%) of them had low birthweight (< 2500g) and 13 (3.9%) were pre-term (10 < 37 weeks; 03 < 32 weeks). As for the other group, we had 88 multiple pregnancies (85 twins and 3 triplets) resulting from the transfer of 215 embryos. Of the twins pregnancies, 7 resulted in abortion (5 from DET, 1 from TET and 1 from QET), and so we had 162 newborns from multiple pregnancies: 2 newborns (1 pregnancy) from SET (monochorionic), 76 newborns (38 pregnancies) from DET, 50 newborns (25 pregnancies) from TET and 6 newborns (3 pregnancies) from QET. It is noteworthy that 3 of the twins pregnancies (1 from DET and 2 from TET) had fetal death resulting in only one newborn each. Mean birthweight for multiple pregnancy newborns was 2285,05g ± 397,56, and mean gestational age was 36.58±1.86 weeks; 103 (67.8%) of them had low birthweight (<2500g) and 53 (33.5%) babies were pre-term. Both the birthweight and the gestational age were significant higher in singletons compared to multiple pregnancy newborns (*p*<0.001). When comparing all 131 low birthweight newborns (26.9%), 21.4% were singletons and 78.6% were from multiple pregnancies; comparing all the 66 pre-term babies (13.5%), 19.7% were singletons and 80.3% were born from multiple pregnancies.

**Conclusions:** Literature analyzes mostly twins pregnancies, but we feel the data can be extrapolated to multiple pregnancy. Thus, although our data is in accordance to what is expected - lower birthweight and gestational age in multiple pregnancy newborns comparing to singletons - we noticed we have better results for pre-term newborns. Whereas literature show that the incidence of pre-term multiple pregnancy newborns vary from 43.7% to 61.2%, our occurrence for that is only of 33.5%. Although there was one study that reports a rate of 39.2% of twins pre-term, they considered a cut-off of 36 weeks in instead of the usual 37 weeks. If we considered the same cut-off, our incidence for pre-term babies derived from multiple pregnancies would be 15.8%. On the other hand, our rates for low birthweight are very similar to those found in literature.

**P-50. Clomiphene Citrate x Letrozole: literature review about the success in ovulation induction**

**Isabela Clarassoti Simionato** ^**1**^ **, Isabella Teruko Matsuyama** ^**1**^ **, Isadora Germano Fernandes Bastos** ^**1**^ **, Gabriel Monteiro Pinheiro** ^**2**^

^*1*^ *Universidade de Santo Amaro.*

^*2*^ *Disciplina de Ginecologia e Obstetrícia da Universidade de Santo Amaro.*

**Objective:** Provide theoretical framework about the drugs Clomiphene Citrate and Letrozole, comparing them in terms of effectiveness and live birth rate.

**Methods:** Literature review, with search in BVS, MEDLINE and PUBMED databases, including cohort studies, case-control studies, randomized clinical trial, clinical practice guide, master's and doctoral theses and systematic review, between the years 2010 to 2020. Case reports were excluded.

**Results:** Infertility is a growing challenge in Brazil, due to the choice of women in getting pregnant later. Therefore, it is important to discuss the available therapeutic options. The manuals use Clomiphene Citrate (CC) as the first line of treatment for ovulation induction, but other countries have been using Letrozole exhaustingly, a drug prescribed as "off-label" in Brazil. CC has a weak estrogenic effect, stimulating ovulation by releasing GnRh and gonadotropins. Letrozole, on the other hand, is an aromatase inhibitor, used by CC-resistant women, which acts by increasing follicular sensitivity to FSH. In comparison, it was observed that the ovulation rate of letrozole and CC did not differ significantly. It was also observed that CC caused an extremely thin endometrial thickness in 50% of women. The rate of pregnancies that did not progress with the use of CC (18.5%) was higher compared to Letrozole (11.5%). In several clinical trials, women who received letrozole had higher live birth rates compared to CC. 

**Conclusion:** Letrozole is as effective as CC, especially in ovulation and live birth rates, plus it demonstrated superiority in terms of endometrial thickness and gestational evolution. Hence, this drug should be considered a good option for ovulation induction.

**P-51. Evaluation of abandonment rate of cryopreserved sperm in a private semen bank**

**Tiago Cesar Mierzwa** ^**1**^ **, Debora Scaraboto** ^**1**^ **, Rosana Bueno de Moraes Hernandes** ^**1**^ **, Lidio Jair Ribas Centa** ^**1**^

^*1*^ *Andrology Department of Fertility Clinic - Androlab; Curitiba - PR.*

**Objective:** Semen freezing is a specialized technique used for patients undergoing gonadotoxic treatment. For men it is a simple procedure that involves collecting the sample on the day of the freeze and paying a maintenance fee, usually charged semi-annually or annually. It is increasingly common for banks to store cryopreserved sperm from patients who have no longer made contact nor payed the fees. These sperm are considered "abandoned". The aim of this study was to analyze the abandonment rate in a private semen bank.

**Methods:** This is a retrospective study. In the studied sperm bank, the maintenance fee is charged every six months. Abandonment was considered non-payment of maintenance fees from patients lost to follow up for a period of at least one year. The period from 1980 to 2020 was analyzed and 1007 patients who cryopreserved spermatozoa were included.

**Results:** 82 patients were included in the non-paying group, making up an abandonment rate that corresponds to 6%. Stratifying these patients for the reason of freezing, it can be analyzed that 43% were cancer patients who cryopreserved before chemotherapy or radiotherapy treatments, 17% were patients undergoing ICSI, IUI or IVF procedures who cryopreserved because they could not be present on the day of procedure and 11% frozen pre-vasectomy semen, among other reasons with lower percentages.

**Conclusion:** Assessing the dropout rates, it can be noted a large number of patients who froze the semen before cancer treatments, forming the hypothesis that the lack of communication is due to the worsening of the disease or the patient's death. Another large portion are patients who cryopreserved before undergoing assisted reproduction treatments, and success in the treatment may have been the reason for abandoning the cryopreserved gametes.

**P-52. Uterine Transplantation - Retrospective and what's new in the Brazilian scenario**

**Isabela Clarassoti Simionato** ^**1**^ **, Barbara Neudine Sandrini Baptista** ^**1**^ **, Gustavo Nóbrega Barbosa** ^**1**^ **, Leticia Vezneyan Povia** ^**1**^ **, Thays Nolasco** ^**1**^ **, Gabriel Monteiro Pinheiro** ^**2**^

^*1*^ *Universidade de Santo Amaro.*

^*2*^ *Disciplina de Ginecologia e Obstetrícia da Universidade de Santo Amaro.*

**Objective:** This literature review aims to evaluate the retrospective scenario of uterine transplantation and where the Brazilian human reproduction is currently located.

**Method:** Literature review, based on articles collected in Pubmed, BVS and SciELO databases, published between 2016 and 2020 in Portuguese and English.

**Results:** Uterine transplantation (UTx) is a surgical-therapeutic option for patients with uterine malformations, previous hysterectomy, and non-surgically correctable intrauterine adhesions. Historically, the first UTx was from a live donor, performed in Saudi Arabia in 2000, however it was without success due to acute vascular thrombosis, requiring a hysterectomy three months after surgery. The second UTx was performed in 2011 in Turkey, where two pregnancies were reported, althugh both were unsuccessful due miscarriage. The transplant that culminated in the first child born alive, with an uterus also received from a living donor, was in Sweden in the year 2014. In Brazil, according to the World Health Organization, around 8 million people may be infertile, increasing the desire to search for alternative forms of pregnancy. In this scenario, a motivation emerged to start the preliminary study in sheep, due to its anatomical similarity to women; four successful auto transplants were performed, being the first two in 2016, with the aim to experiment and standardize the technique. After the success achieved, a safer and more technically oriented space was opened for carrying out an experimental procedure in women apt to do it. Consequently, a 32 years old woman with Mayer-Rokitansky-Küster-Hauser underwent the world's first uterine transplant from a dead donor. The donor was 45 years old, with a history of three previous vaginal deliveries and died of subarachnoid hemorrhage. The surgery was successful, and the organ received uneventfully. The receptor menstruated after 37 days, the pregnancy occurred after the first transfer of a single embryo, 7 months after transplantation, at the end of the 36th week a cesarean section was performed, accompanied by hysterectomy and suspension of immunosuppressants, the female baby weighed 2,550 g at birth, suitable for gestational age, with Apgar scores of 9 in 1 min, 10 in 5 min and 10 in 10 min, and along with the mother, she remains healthy and developing normally 7 months after delivery. This UTx become an important mark for human reproduction. However, not only family consent is a great barrier in many countries, but also, there are other impediments, such as religious concerns, costs, tissue viability and the availability of trained professionals for this procedure.

**Conclusion:** Since the technique is rising constantly in Brazil, uterine transplantation has been considered a safe therapeutic option for uterine infertility, enabling women with the previously mentioned conditions to have a pregnancy that is not only viable, but also healthy, as previous studies demonstrated favorable outcomes. Furthermore, considering that UTx can be performed with living and deceased donors, the possibility of receiving a compatible uterus and achieving the desired pregnancy becomes more feasible. However, for this procedure to take place, especially trained and qualified professionals are needed. In addition, a specific legislation has an imperative need to be established, as the number of successful cases has led to further expectations on performing this technique widely in specialized centers, overcoming its experimental stages into a common procedure for those in need.

**P-53. Correlation between specific times of embryo development of a time-lapse cultivation system with increase in clinical pregnancy - Retrospective study**

**Andrea Mesquita Lima** ^**1**^ **, Gleicyane Sousa dos Santos** ^**1**^ **, Sebastião Evangelista Torquato Filho** ^**1**^ **, Eduardo Gomes Sá** ^**1**^

^*1*^ *BIOS - Centro de Medicina Reprodutiva do Ceará.*

**Objective:** Time-lapse culture systems have provided great advances in relation to embryo culture. As it is a technology in which it is possible to observe cell divisions in real time, making it feasible to correlate morphokinetic parameters with several findings, including clinical pregnancy rates. The study aims to analyze how the times of disappearance of the pronuclei (tPNf), morulation (tM) and blastulation (tB) can affect the clinical pregnancy outcomes of patients undergoing an assisted reproduction program.

**Methods:** Retrospective study that analyzed specific times of embryos cultured in an incubator with the time-lapse system (EmbyoScope+^®^) of 132 patients, from March 2019 to December 2020. Only embryos that presented all markers of the times chosen for the study, tPNf, tM and tB. All embryos were cryopreserved and later transferred.

**Results:** In 2019, patients with positive beta presented the following results for each time analyzed: tPNf (23.22h), tM (89.46h) and tB (111.19h). Meanwhile, patients from 2020 who presented positive Beta had the following results: tPNf (22.75h), tM (85.98h) and tB (105.48h). It was possible to observe, by comparing the times of positive beta patients with negative betas in the year 2019, that there were no significant differences. However, there was a slight disparity in the morulation times between beta-positive patients (tM = 85.98h) and beta-negative ones (tM = 92.34h).

**Conclusion:** After analyzing the times mentioned and comparing them with the clinical pregnancy results, it is possible to predict that some times analyzed by the time-lapse system may slightly increase the clinical pregnancy rates, as it was possible to observe, for example, when the time of morulation is less than 90h of culture, it may be indicative of a positive beta result.

**P-54. Pregnancy losses: the importance of the therapeutic space for the legitimization of grief and new pregnancies**

**Aline Borges de Araújo** ^**1**^ **, Juliana Roberto dos Santos** ^**2**^

^*1*^ *Private Office.*

^*2*^ *Ideia Fertil.*

**Objective:** The loss of a child before birth is a neglected loss, not accepted, despite fetal death and especially abortion, being a possibility and, according to medicine, common. What is not taken into account is the suffering and helplessness, especially of mother who experiences such loss. For Iaconelli (2007) “we anticipate the understanding of this mourning as something unusual, since its characteristics present unusual aspects that make it incomprehensible and unrecognizable by the surroundings”. The exclusion of such suffering hampers the viability of portraying it, there is no dead child. It is noted that for women who use assisted reproduction techniques, fear of fetal death and anxiety are present during pregnancy, often due to the difficulty of success or numerous treatments, as well as poorly elaborated mourning, such as loss of the ability to get pregnant naturally. It is also perceived that abortion can discourage them from trying a new treatment, causing feelings of worthlessness and incapacity, as stated by Quayle (2020).

**Methods:** In order to outline what was lost, it is important to recognize that the mother lost the dreamed baby and it was this child who was being libidinally invested. With the arrival of the newborn, the mother lives with the real baby and thus loses the supposed baby. In loss, the mother seeks recognition for the lost child and for the people around her, it is difficult to imagine such pain. Some fathers/mothers prefer to perform a kind of ritual to deal with the pain, however, this ceremony does not guarantee the elaboration of what was not done. The ideal model is that mother can deal with the imposed reality and that her suffering can be socially legitimized.

**Results:** People who imagine that the pain of the loss of a child will depend on the time spent together, will not be able to agree on the grief of a child “so small”, “still embryonic” as some would say, time gives the idea of greater bonding. In fact, the ties between parents and children are tightened with the routine, what is not discussed in the loss of an intrauterine baby is that the mother establishes an emotional and idealized bond with her baby and this feeling must be considered. The mother must be made to work out her grief. For Iaconelli (2007), the mother is doubly helpless: by the baby and by the adults. Health professionals who take care of these women must consider psychotherapy as an ally, which legitimizes the suffering of the loss of the child and helps in the elaboration of such loss, the mental health professional is able to identify whether it is a pathological grief or not. The mother must have a space to express her pains and affections, fantasies and frustrations. In order for a woman to be able to invest libidinally in something in life, she will need to disconnect from the lost object. The listening offered to mothers/fathers must be sensitive and consider that each subject/couple will experience this loss in their uniqueness.

**Conclusion:** Men and women usually experience this grief in very different ways, and due to the lack of understanding about the partner's experience, many abortions end up having repercussions even in marital life. While it is certainly a narcissistic pain, abortion is not an irreparable pain. The analysis, when performed after the first pregnancy loss, allows the woman to invest more freely in a new pregnancy. Through the symbolization that the analyst's listening makes possible, the woman can give new meaning to previous experiences and invest in a new object, opening the way for this child to be born and reborn to its mother, which will bring benefits to the woman, the baby and the whole family (Freire & Chatelard, 2009).

**P-55. Parthenogenesis in Assisted Human Reproduction**

**Daniela Fink Hassan** ^**1**^ **, Karina Reinaldo Fattori** ^**1**^

^*1*^ *Wahib Hassan Human Reproduction Center. Penápolis/SP.*

**Objective:** We define parthenogenesis as a process of formation of a haploid gamete in a diploid cell, without any fertilization process of sexual reproduction. It is a reproduction strategy in vertebrate species in which no sperm are involved in embryonic development from the oocyte. The study describes a case report of spontaneous parthenogenesis in human oocytes from a cycle of ovarian stimulation for in vitro fertilization in an elderly patient with low ovarian reserve (Poseidon group 4).

**Results:** Ovarian stimulation was performed with ultrapurified gonadotropins at a dose of 300 IU daily for a period of 13 days, using an antagonist and dual trigger protocol. As a result of ovarian stimulation we obtained a total of two retrieved oocytes evaluated according to the oocyte morphological classification as parthenogenic. Parthenogenetic oocytes revealed that, at the time of recovery, all exhibited cytoarchitectural characteristics oocytes similar to those present in a fertilized oocyte, influencing the structural and morphological characteristics of the recruited oocytes. The etiology of this fertilization accident called parthenogenesis would be unpredictable and the presence of a detailed genetic diagnosis in the result of parthenogenic oocytes is rarely available, it is not clear the genetic alterations found and the evolution of embryonic development in humans. However, it would be possible in some cases in assisted reproduction the spontaneous activation of the oocyte leading to parthenogenesis is related to the treatment of ovarian stimulation with hormones and protocols influencing the structural and morphological characteristics of the recruited oocytes.

**Conclusion:** Faced with the possibility of recurrence of the oocyte parthenogenesis phenomenon and the low chances of pregnancy with own oocytes due to advanced age, the team at the reproduction center indicated an ovoreception program as a treatment option.

**P-56. Egg maturation arrest in *In Vitro* Fertilization cycle: “Poor Quality Oocyte Syndrome”**

**Talita Colombo** ^**1**^ **, Adriana Arent** ^**1**^ **, Isadora Badalotti-Teloken** ^**1**^ **, Catarina H Petracco** ^**1**^ **, Rafaella Petracco** ^**1**^ **, Alvaro Petracco** ^**1**^ **, Mariangela Badalotti** ^**1**^

^*1*^ *Fertilitat - Medicine Reproductive Center - Porto Alegre - RS.*

**Objective:** The oocyte maturation process comprises many structural and molecular alterations in the cytoplasm (cytoplasmic maturation). It results in a chromosomal configuration in metaphase II (nuclear maturation), and it precedes the sperm penetration and its activation to fertilize. The maturation arrest in human oocytes can occur at different stages of the mitosis cycle. The total failure on completing meiosis is rarely found in *in vitro* fertilization cycles (IVF).

**Results:** A couple presenting unexplained infertility for four years - the woman with 35 years old and her partner with 37 years old - was referred to an IVF treatment. The ovarian reserve was evaluated by measuring the anti-Mullerian hormone (1,41 ng/ml) and antral follicle count (eight). The ovarian stimulation was carried out with human menopausal gonadotrophin (totalizing 2425 UI); 0,25 mg of gonarelix was used during eigth days, from day eight. There were 13 follicles > 16 mm in diameter. When at least three follicles had a diameter greater than 17 mm, 250 mg of recombinant hCG was administered for triggering, and follicular aspiration was performed 35 hours later by transvaginal ultrasound. Thirteen follicles were aspirated, nine eggs were retrieved, and all were immature in the germinal vesicle stage or prophase I (P1). The oocytes were left in a culture medium for in vitro maturation with no success. A new IVF cycle was performed using FSH + recombinant LH stimulation during three days (600 UI) + human menopausal gonadotrophin during 12 days (2136 UI), with a good ovarian response - nine follicles > 16 mm; 0,25 mg of gonarelix was used during five days, from day eleven; double triggering (250 mg recombinant hCG + 0,2 mg triptorelin) was performed for follicular maturation. However, all of the nine oocytes retrieved were immature in stage P1.

**Conclusion:** Approximately 20 to 30% of the oocytes collected in IVF cycles are meiotically immature at retrieval, probably due to the stimulation of multiples follicles. When the percentage of immature oocytes is above 25%, the results of IVF are negatively affected. The occurrence of a total failure of oocyte maturation in IVF cycles is not common in the literature. The failure of oocyte maturation can cause infertility in couples that once had been diagnosed with unexplained infertility. The recognition of oocyte maturation as a specific factor of infertility demonstrates the importance of this factor in reproductive medicine. In this case, all the oocytes stopped development at the germinal vesicle stage. In this situation, the use of donated eggs must be considered since even with different stimulation protocols is not possible to obtain mature eggs.

**P-57. Comparison of results in ovarian stimulation cycles using dydrogesterone and GnRH antagonist**

**Agnaldo Viana** ^**1**^ **, Genevieve Coelho** ^**1**^ **, Danielle Freitas** ^**1**^ **, Matheus Fér Pereira** ^**1**^

^*1*^ *Instituto Valenciano de Infertilidade (IVI), Salvador, Bahia, Brasil.*

**Objective:** About 186 million people, approximately 15% of the world's couples, live in a context of infertility. Many patients are indicated to in vitro fertilization as a solution for infertility. Ovarian stimulation figures as one of the central stages of infertility treatment and preservation of female fertile potential. One of the challenges during ovarian stimulation during IVF is inhibition of premature LH peak and its consequent early ovulation, which occur in response to the increasing concentration of estradiol during the cycle. The use of progestins in the prevention of LH peak was suggested with the hypothesis elaborated by Letterie et al. Noretindrone, medroxiprogestrona and dydrogesterone were successfully used for this purpose. Our study aims to evaluate the use of dydrogesterone compared to GnRH antagonist in IVF cycles. We will evaluate the number of oocytes, maturation rate, fertilization and pregnancy in each cycle.

**Methods:** We conducted a retrospective study with the cycles conducted in our center (Valencian Institute of Infertility, Salvador, Bahia, Brazil) between January 2017 and December 2019. All patients were egg donors, aged between 18 and 35 years, without comorbidities and selected after screening tests for fertility, serological and genetic. The cycles were performed with stimulation with daily doses of 150-225 uni of FSH + LH, trigger with GhRH analogue. Cycles with dydrogesterone were used daily dose of 10mg from the first day of stimulation until the last day of the cycle. Antagonist cycles, this was introduced when the largest follicle mean 14mm of average diameter. Cycles with poor response, or with oocytes intended for vitrification, were excluded from this study. Categorical variables were described as frequencies and percentages. The relationships between the variables were described using the odds ratio (OR) as the mean of association of confidence intervals (95% CI) and bivariate analyzes were tested using the chi-square test or Fisher's exact test. Continuous variables with normal distribution were described as mean standard deviation and nonnormal variables as median and interquartile range (IIQ) of Mann-Whitney. Values of P< 0.05 were considered statistically significant. The analyzes were performed using the Statistical Package for the Social Sciences (SPSS version 20.0) program.

**Results:** Between 2017 and 2019, 214 cycles of ovarian stimulation of donors were recorded. Of these, dydrogesterone was used in 69 cycles, while the GnRH antagonist was used in 145. The general characteristics of the sample are described in Table 1, with average age, number of follicles recruited to USG, number of oocytes recovered and number of mature oocytes. Regarding the outcomes analyzed in the bivariate analysis with percentages of mature, arrival rate at blast and rate of pregnancy and abortion, are described in table 2.

**Table 1.** General characteristics of the sample according to the drug used.

Variable	Duphaston (n=69)	GnRH Antagonist (n=145)	*P*-value
Age Average (DP)	27.10 ± 4.27	24,88 ± 3,90	<0.001^a^
Follicles (>14) Average (DP)	17.52 ± 6.01	18.03 ± 6.34	0.444^a^
Recovered eggs Median (IIQ)	17.0 (12.0-25.0)	19.0 (14.0–26.0)	0.274^b^
Mature Median (IIQ)	13.0 (9.0–19.0)	15.0 (11.0–21.0)	0.101^b^

a t-test of Independente Sample;

b Mann-Whitney test;

**Table 2.** Bivariate analysis according to the drug used.

Variable	Duphaston (n=69)	GnRH Antagonist (n=145)	*P*-value	OR (IC = 95%)
Mature rate (%) Median (IIQ)	80.0 (66.7-88.9)	80.0 (69.6-92.3)	0.459^b^	-
Fertilization rate (%) Median (IIQ)	80.0 (70.0-100.0)	75.0 (60.0-87.5)	0.003^b^	-
Blastocyst-stage ratio (%) Median (IIQ)	66.7 (50.0-83.3)	57.1 (33.3-72.7)	0.022^b^	-
Pregnancy, n (%)	42 (60.9)	86 (59.3)	0.828^c^	1.06 (0.59 - 1.91)
Twin Pregnancy, n (%)	8 (11.6)	33 (22.8)	0.052^c^	0.44 (0.19 - 1.04)
Clinical abortion, n (%)	4 (5.8)	15 (10.3)	0.274^c^	0.53 (0.17 - 1.67)

b Mann-Whitney test;

c Pearson’s Qui-square test; d Fischer’s Exact test.

d Fischer’s Exact test.

**Conclusions:** In the study observed we have the same rate of mature oocytes when we performed hypophistory inhibition with dydrogesterone and GnRH antagonist. However, pregnancy and abortion rates were similar in both groups observed.

**P-58. PGT-A for all? Challenging the global trend, challenges and criticism when there is no indication**

**Gabriela Gouveia** ^**1**^ **, Sylvia Sanches Cortezzi** ^**2**^ **, Isabella Teruko Matsuyama** ^**1**^

^*1*^ *Universidade Santo Amaro.*

^*2*^ *Instituto Idéia Fértil de Saúde Reprodutiva, INSTITUTO IDÉIA, Brasil.*

**Objective:** Preimplantation genetic testing (PGT) enables a chromosomal or genetic analysis of a developing embryo, by biopsying one or more cells, before transfer to the mother's uterus. The purpose of diagnosis is to select chromosomally and/or genetically normal embryos. For patients with advanced age, repeated abortions, altered karyotype, between others, the diagnostic method used is the Preimplatation Genetic Test for Aneuploidies (PGT-A), for investigation of aneuploidies. The main point of reflection on the PGT-A is the possible disposal of mosaic embryos, which could be compatible with life, or even diagnostic errors that can lead to the discard of healthy embryos. Objective: Analyze the use and conditions for PGT-A indications.

**Methods:** This is a literary review using the descriptors: "PGT-A" and "Indication" searched in the PUBMED database, with filter between the years 2011 and 2021.

**Results:** In a 2015 study, 10.852 cycles with 58.798 embryos were evaluated, showing that aneuploidies increase significantly after the maternal age of 35 years, and the in vitro fertilization (IVF) success rate decreases due to oocyte quality. The higher the maternal age, the lower the rate of chromosomally normal embryos, reaching 13% in women older than 42 years. The rate of live births resulting from the transfer of aneuploid embryos is 0%, a mosaic of up to 40% of altered cells has a rate of live births of 15% to 40%, in the case of euploid embryo transfer the rate of live births is 40% to 68%. The agreement rates found between biopsies and culture media were 80-85% in the D6 collection. Genetic counseling is essential to know the couple's history, help the individual or family to understand the aspects involved, including the diagnosis, the likely course of the disease and the available management. In this technique, embryos that have some chromosomal alteration are considered unviable and are discarded or donated for research.

**Conclusion:** Even if there are indications for the use of the test, it is important that there is genetic counseling, so that the patient is aware of the benefits, risks and limitations of genetic testing, it is also necessary to discuss the ethical issues involved in the technique, as it is still far away the possibility of having a consensus on them. It is believed that, in the future, the development of less and less invasive techniques involved in pre-implantation genetic tests may allow a more accurate and reliable diagnosis.

**P-59. Progestin-primed ovarian stimulation with Dydrogesterone versus GnRH antagonist on oocytes and embryo results on controlled ovarian stimulation cycles**

**Marcelo Lucchesi Montenegro** ^**1**^ **, Edson Guimarães Lo Turco** ^**1,2,3**^ **, Fernanda Rodrigues Bernarde** ^**2**^ **, Silvia Morales Jau** ^**1**^ **, Amanda Lino de Faria** ^**1**^ **, Fernando Prado Ferreira** ^**1,2**^

^*1*^ *Neo Vita.*

^*2*^ *UNIFESP.*

^*3*^ *EmbrioLógica.*

**Objective:** Progestin-primed ovarian stimulation (PPOS) is an emerging protocol in which oral progesterone is administered during controlled ovarian stimulation (COS) in order to prevent premature LH surge. This study intends to compare laboratory and clinical outcomes of COS using dydrogesterone (DYG) or GnRH antagonist to prevent premature LH surge in women undergoing in vitro fertilization (IVF) cycles.

**Methods:** We performed a retrospective cross-sectional study including 25 patients. They were separated into two groups. The first group (n=11) received recombinant follicle-stimulating hormone (FSHr) associated with DYG from the third day of the menstrual cycle; the second group (n=14) received FSHr starting at the beginning of the menstrual cycle and GnRH antagonist (Orgalutran) was added after the leading follicle achieved 14-15 mm of diameter. Both groups were submitted to oocyte retrieval 35 hours after GnRH agonist, administered when the larger follicle reached 19-20 mm. Patients who intended to become pregnant had their embryos cryopreserved for a later uterine transfer cycle. The number of follicles, oocytes, and embryos were compared between the two groups, as well as the total dose of FSHr and total cost of medication on each protocol.

**Results:** There were no significant differences in patients' age, AMH levels, and time of infertility between the two groups. Compared with GnRH antagonist group, the number of follicles larger than 14 mm on the day of trigger (9.91±9.24 *vs*. 9.43±6.75, *p*=0.88) and a total number of oocytes retrieved (9.91±8.5 *vs*. 12.57±9.81, *p*=0.48) were similar on the DYG group, as well as the number of metaphase II (MII) (6.27±5.46 *vs*. 7.5±5.77, *p*=0.59) and immature oocytes (2.64±2.54 *vs*. 3.64±3.47, *p*=0.42). Regarding embryo results, no significant differences were found in the number of day 3 embryos (5.64±5.67 *vs*. 5.79±4.79, *p*=0.94) or blastocysts viable for freezing (3±2.89 *vs*. 2.07±1.9, *p*=0.34). Analyzing the drugs using during the stimulation, the total dose of FSH was equivalent in the groups (2727.27±629.52 *vs*. 2785.71±602.05, *p*=0.81), but the number of days with a LH suppressor were larger in DYG group (10.11±1.16 *vs*. 4±1.03, *p*<0.05).

**Objective:** DYG can be considered an option to prevent premature LH surge on controlled ovarian stimulation IVF cycles, with no differences in the number or quality of oocytes and embryos available for a later transfer. A longer period of LH blockage during COS does not interfere with those results.

**P-60. The Level of Knowledge About Reproductive Health Among Patients Seen at an Infertility Unit of an University Hospital**

**Fernanda Polisseni** ^**1**^ **, Thais Melo Pereira** ^**1**^ **, Ana Julia Rodrigues** ^**1**^ **, Julia Gabetto Nascimento** ^**1**^ **, Marina Aquino Marge** ^**1**^

^*1*^ *Universidade Federal de Juiz de Fora.*

**Objective:** Infertility is a condition of increasing prevalence in the global scenario. This can be associated with several factors, such as advanced female age, inappropriate nutritional habits, sedentary lifestyle, gynecological pathologies and male factors. Women with reproductive desire must receive correct information regarding fertility preservation, factors that directly influence their reproductive capacity, and the ideal moment to seek medical help. The present study aims to recognize the level of knowledge of patients seen at an University Hospital in Juiz de Fora, Brazil, regarding reproductive health, allowing a better educational approach aiming the most frequent doubts and myths about the subject.

**Methods:** Cross-sectional study in which questionnaires containing 10 polar questions about women's knowledge of reproductive health were applied while they were waiting for a medical appointment in the waiting room of the infertility unit at an University Hospital . Sixty-one women, aged over 18 years, participated in the study between August 2019 and September 2020. The answers were distributed in tables, in which the number of negative answers per question was evaluated, as well as the weighted mean of these answers by age group and education to verify if there were significant differences in the level of knowledge about infertility in these groups.

**Results:** Among the 61 patients who participated in the study, 21.31% had a first degree, 55.54% had completed high school and 22.95% had a college degree. An important relation was established between the level of education and the lack of knowledge about infertility and reproductive health issues among the participating women. There were about twice as many negative answers (lack of knowledge) to the questions asked when the comparison was made between women with first and higher education (15.38% versus 7.14%). Topics such as the relation between smoking and alcohol consumption and infertility, the importance of a balanced diet and physical activity for reproductive health, knowledge about egg freezing for fertility preservation, among others, were addressed. In this context, the question that presented the highest number of negative answers, with 22.95%, was about the comprehension that the time considered normal for pregnancy to occur, for a sexually active couple, without contraception, would be one year. Meanwhile, the highest index of knowledge, with 96.72% of positive answers, was concerning the reduction of chances of pregnancy with advancing age. In the analysis regarding the age pattern of the participants, it was evidenced a lower degree of knowledge in the extremes of age (between 18-24 years and in those older than 60 years), which can be due to several factors, including the deficient sexual and reproductive education and the sociocultural context in which they are inserted.

**Conclusion:** Based on the data obtained, we noticed that a significant portion of women does not know relevant information about infertility. There also is an inversely proportional relation between education level and level of knowledge on the subject, which alerts us to the inequality of access to knowledge among these segments. About the data related to age and level of knowledge, it is disturbing that women aged 18 to 24 are among the least informed since this is an important age to intervene in lifestyle factors, such as regular physical activity and healthy eating It is of utmost importance to offer information on reproductive health, in a clear and accessible way, for women, so they could know the right time to seek medical help and could have more self-knowledge, leading the care of their health.

**P-61. Assessing the impact of smoking on seminal parameters**

**Tiago Cesar Mierzwa** ^**1**^ **, Debora Scaraboto** ^**1**^ **, Andressa Thais Culpi** ^**1**^ **, Lidio Jair Ribas Centa** ^**1**^

^*1*^ *Andrology Department of Fertility Clinic - Androlab; Curitiba - PR.*

**Objective:** Smoking is a habit that can result in damage to sperm characteristics since inhaled toxins can alter gametogenesis and the functionality of male gametes. However, recent studies showed no changes in sperm parameters of smokers patients compared to non-smokers. The aim of this study is to investigate the association between smoking and the seminal parameters of volume, concentration, motility and morphology in patients who sought a fertility clinic for a spermogram exam.

**Methods:** Retrospective study based on the analysis of surveys from 1393 patients who underwent sperm analysis in a private fertility clinic in Curitiba between 2017 and 2021. The parameters assessed were: total ejaculate volume, concentration (sperm/mL), progressive motility and morphology, based on WHO guidelines from the Laboratory Manual for the examination and processing of human semen (5th edition). The values considered normal were: seminal volume ≥1.5mL, concentration ≥15million sperm/mL, motility ≥32% of progressive motile sperm and morphology of at least 4% normal sperm (Kruger's strict standard). Varicocele, vasectomy or vasectomy reversal patients or those who have or have had cancer were not included in the study.

**Results:** 11% (n=158) of all patients reported being smokers and 89% (n=1235) reported not smoking. Among the Non-smokers group, 6.7% (n=83) had abnormal ejaculated volume, 20% (n=247) had lower concentration, and 2% of the total showed azoospermia. 37% (n=461) had asthenozoospermia and 50% (n=612) had lower sperm morphology. In the Smokers group, 10.76% of the patients had changes in ejaculate volume (n=17), 23% (n=36) reduced concentration, 3% showing azoospermia, 39% (n=61) low motility and 47% (n=75) teratozoospermia. There was no statistically significant difference between the parameters when comparing the two groups (volume *p*=0.062, concentration p=0.5398, motility *p*=0.6244, morphology *p*=0.4776). Statistical analysis was calculated using the two-tailed chi-square test with a significance level of 0.05.

**Conclusion:** There was no statistical difference between the parameters analyzed for the "Smokers" and "Non-smokers" groups. The fact that the patients analyzed came from a fertility clinic (despite not always undergoing Assisted Reproduction Techniques) may cause a greater number of infertile patients from various other causes to be included in the "Non-smokers" group, which could affect the study results. A more advanced study should only consider patients not investigating infertility and, therefore, our questionnaires were revised to include this information. We hope in future studies to be able to develop this research again only considering these patients.

**P-62. Viability of blastocyst formed from abnormal zygotes**

**Christina Kelly Machado Holanda** ^**1**^ **, Andrea Mesquita Lima** ^**1**^ **, Gleicyane Sousa dos Santos** ^**1**^ **, Sebastião Evangelista Torquato Filho** ^**1**^ **, Fábio Eugênio Magalhães Rodrigues** ^**1**^ **, Marcus Aurélio Bessa Paiva** ^**1**^ **, Tulius Augustus Ferreira de Freitas** ^**1**^

^*1*^ *BIOS - Centro de Medicina Reprodutiva do Ceará.*

**Objective:** In assisted reproduction techniques, sperm can be directly inserted into the cytoplasm of mature oocytes, through intra-cytoplasmic sperm injection (ICSI). After ICSI, sperm stimulate the egg to complete the second meiotic division. From then onwards, the condensation of the maternal chromosomes takes place, and the oocyte nucleus becomes the female pronucleus. The sperm nucleus enlarges inside the oocyte aiming to compose the male pronucleus. The occurrence of fertilization can be confirmed with the appearance of two pronuclei: a female and a male. However, in some situations, an abnormal fertilization can occur, where only one pronucleus or more than two can be seen. The objective of this work was to analyze blastocysts from zygotes with only one pronucleus, and which had cells sent for genetic analysis and its relationship with embryonic aneuploidy.

**Methods:** From January 2020 to July 2021, zygotes with only one pronucleus were analyzed and kept in culture until the blastocyst stage. Of those who reached the blastocyst stage, 13 underwent embryonic biopsy, and had cells sent for genetic analysis

**Results:** Of the 13 blastocysts that had their cells genetically analyzed, 03 had normal results, that is, they were euploids, according to the genetic analysis report, 04 had embryonic aneuploidy, 03 had low-grade mosaic and 02 had no detectable DNA for analysis.

**Objective:** A fertilization with only one pronucleus is considered abnormal, and may be due to an artificial activation of the oocyte or failure in the development of the maternal or paternal pronucleus. However, despite a low number analyzed, it is clear that such zygotes can generate genetically normal embryos, and viable for transfer to the mother's uterus.

**P-63. Effect of weight loss guidance on ovulation of obese and overweight infertile patients**

**Beatriz Bacheschi do Carmo Benetti** ^**1**^ **, Mário Silva Approbato** ^**2**^ **, Fabiana Carmo Approbato** ^**3**^

^*1*^ *Laboratório de Reprodução Humana HC/UFG.*

^*2*^ *Centro de Reprodução Humana - Departamento de Obstetrícia e Ginecologia da Universidade Federal de Goiás, Brasil.*

^*3*^ *Universidade Federal de Goiás, Brasil.*

**Objective:** To evaluate the effect of counseling on weight reduction on ovulation in obese and overweight infertile patients.

**Methods:** Retrospective cohort. In this study, an evaluation of the weight loss of a population of infertile patients with overweight and obesity was carried out in a period of out one to two years after the first consultation. Next, the parameters were compared with the data from the first medical appointment. In the first consultation at the assisted reproduction clinic (Human Reproduction Laboratory HC/UFG), obese and overweight patients were instructed to lose weight and informed that being overweight could reduce the chances of being successful in the treatment. Patients were matched for age and endocrine variables (Table 1). The statistic applied was the Chi-square test.

**Table 1.** Distribution of patients, according to confounding variables (age, FSH, LH and estradiol) - Human Reproduction Laboratory HC/UFG, 2021.

Variable		BMI normal			BMI overweight and obesity		
	n	x	Sd	n	x	Sd	*p*
Age	44	35.59	3.23	56	35.78	3.70	0.3916
FSH	44	7.36	3.37	56	6.42	2.72	0.0635
LH	44	5.46	4.66	56	8.40	17.11	0.1114
Estradiol	44	58.55	44.23	56	61.55	53.80	0.3831

FSH: Follicle Stimulating Hormone; LH: Luteinizing Hormone; x: mean; Sd: standard deviation.

**Results:**Table 1 shows that the patients were effectively paired with regard to confounding variables.

**Conclusion:** Advising weight loss in infertile overweight and obese patients had no effect on weight reduction. On the contrary, the mean weight of patients after guidance was statistically higher than the mean in the first consultation (both weight and BMI). Regarding ovulation, there was no statistically significant difference between patients who lost and gained weight (Table 2).

**Table 2.** Distribution of patients according to ovulatory status. Comparison between the group that lost weight and the group that gained weight one to two years after the orientation of the first consultation - Human Reproduction Laboratory HC/UFG, 2021.

	Lost weight n (%)	Gained weight n (%)	Total
**Ovulation**	13 (68.4)	20 (80.0)	33
**Anovulation**	06 (31.6)	05 (20.0)	11
**Total**	19	25	43

* Statistical test: chi-square. RR= 0.7222; *p*= 0.5981

**P-64. Evaluation of oocyte morphology in old-age women**

**Karina Reinaldo Fattori** ^**1**^ **, Daniela Fink Hassan** ^**1**^

^*1*^ *Wahib Hassan Human Reproduction Center. Penápolis/SP.*

**Objective:** Maternal age has been considered an impacting factor in the outcome of assisted reproduction (AR) treatment. With aging, there is a rapid decline in the quantity and quality of oocytes. The objective of AR laboratories is to select the best gamete and embryo for transfer, reactivating the concern with oocyte quality. The main objective of this retrospective study was to describe the number of oocytes obtained during ovarian stimulation cycles for the in vitro fertilization (IVF) procedure according to the age group of the women analyzed, describe the incident oocyte morphological changes and establish an association with age, to correlate oocyte dysmorphisms according to age group and to analyze the impact of the age factor in the alteration of the oocyte morphology of these women. The evaluation of oocyte morphology (OM) becomes a difficult task, as the underlying mechanisms that alter the appearance of the oocyte are multifactorial and complex. The existence of morphological variations between oocytes can affect developmental competence and embryo implantation potential, resulting from intrinsic factors such as age and genetic defects, or extrinsic factors such as stimulation protocols, endocrine disruptors and culture conditions.

**Methods:** This was a retrospective study that included a cohort of 15 women referred for IVF to a Reproduction Center between 2020 and 2021. Data were obtained from the patients' clinical and laboratory records. Women over 35 years of age were considered eligible for study selection and graded according to age. All women at the Center were asked to provide informed consent in order for their data to be used for research purposes. Patients were classified according to age group 35-37, 38-40 and > 40 years and according to OM alterations.

**Results:** We observed a linear association between the number of captured oocytes and the patient's age, 36.36% were between 35-37 years, 33.77% were 38-40 years and 29.87% > 40 years. The study showed that OM abnormalities such as ooplasm granularity, inclusions, zona pellucida (ZP) thickening, vacuoles, and first polar body (PC) fragmentation were predominant in patients aged >40. On the other hand, the most observed morphological changes with increasing age of women were: granulosity in the ooplasm in 46.75% of cases, followed by thick ZP in 18.18% and first fragmented PC in 14.29% of oocytes. We established a linear regression analysis where older women recruited fewer oocytes demonstrating a higher rate of morphological changes in relation to the number of captured oocytes.

**Conclusion:** In conclusion, despite the limitations of the number of cases, maternal age is negatively related to oocyte quantity and quality in IVF cycles. OM abnormalities, such as ooplasm granularity, were predominant in this cohort of women, however they need further studies to assess the real impact of oocyte dysmorphism on IVF results. Suggesting that OM could predict the outcome of in vitro fertilization (IVF) cycles, however due to the variety of experimental models and conflicting results, the available literature fails to attribute a significant impact on fertilization and pregnancy outcomes related to specific oocyte dysmorphisms.

**P-65. Effect of weight loss orientation on BMI in obese and overweight infertile patients**

**Beatriz Bacheschi do Carmo Benetti** ^**1**^ **, Mário Silva Approbato** ^**2**^ **, Fabiana Carmo Approbato** ^**3**^ **, Approbato FC**

^*1*^ *Laboratório de Reprodução Humana HC/UFG.*

^*2*^ *Centro de Reprodução Humana - Departamento de Obstetrícia e Ginecologia da Universidade Federal de Goiás, Brasil.*

^*3*^ *Universidade Federal de Goiás, Brasil.*

**Objective:** To evaluate the effect of counseling on weight reduction in absolute values and on the Body Mass Index (BMI) in obese and overweight infertile patients.

**Methods:** Retrospective cohort. In this study, an evaluation of the weight loss of a population of infertile patients with overweight and obesity was carried out in a period one to two years after the first consultation. Next, the parameters were compared with the data from the first medical appointment. In the first appointment at the assisted reproduction clinic (Human Reproduction Laboratory HC/UFG), obese and overweight patients were instructed to lose weight and informed that being overweight could reduce the chances of being successful in the treatment. Patients were age-matched. The statistic applied was the Student t test.

**Conclusion:** Weight loss guidance in this population had no effect on weight loss. On the contrary, the mean weight of patients after guidance was statistically higher than the mean in the first consultation (both weight and BMI). It is suggested that for obese and overweight infertile patients, in addition to guidance for reduction, an appointment with a nutritionist and/or endocrinologist should be immediately scheduled.

**Table 1.** Distribution of patients according to weight before and after weight loss recommendation - Laboratory of Human Reproduction HC/UFG, 2021.

Pre weight			Post weight			
n	x (Kg)	Sd	n	x (Kg)	Sd	*p*
56	77.11	11.77	56	78.95	12.86	0.0046

* Statistical test: t test, *p*=0.05.

**Table 2.** Distribution of patients according to BMI values before and after weight loss guidance – Human Reproduction Laboratory HC/UFG, 2021.

Pre BMI			Post BMI			
n	x (Kg/m^2^)	Sd	n	x (Kg/m^2^)	Sd	*p*
56	29.69	3.75	56	30.42	4.32	0.0038

* Statistical test: t test, *p*=0.05.

**P-66. Impact of COVID-19 on female and male fertility provided by SARS-CoV-2 connection on ACE2 receiver**

**Milena Rojas Antunes** ^**1**^ **, Caroline Arenales Venna** ^**1**^ **, Isabella Teruko** ^**1**^ **, Gabriel Monteiro Pinheiro** ^**1**^

^*1*^ *Universidade de Santo Amaro.*

**Objective:** Identify the impacts of male and female reproductive function with coronavirus infection (SARS-CoV-2), in addition to exploring the possible role of ACE2 receptors in this process.

**Methods:** Analysis of twelve articles published in English and Portuguese on PubMed and Scielo platforms between April 2020 and April 2021.

**Results:** The SARS-CoV-2 binds to the ACE2 receptor to effect its entry into the body, being able to cause damage to the host’s reproductive system. In the male organism, ACE2 is located in the testicles and when activated by COVID-19 infection, it can cause an unbalance in the testicular environment causing local inflammation and a possible autoimmune attack by sperm, leading to infertility. The direct invasion caused by ACE2 receptors create probably testicular damage, affecting testicular functions by secondary immunological and inflammatory responses. This possibility needs to be investigated through follow-up studies of the reproductive functions of reclaimed male patients. Testicular expression of ACE2 has been shown to be age-related, highest in men with 30 years and lower in their 60 years, suggesting a greater susceptibility of younger men to COVID-19 due to the greater expression of ACE2 receiver. Therefore, in fact, the testis are susceptible to coronavirus, needing to study the effects related to reproduction. When comparing two Chinese studies, Li D et al. 2020 detected the virus in seminal samples from patients with COVID-19 in the acute form of the disease and in the recovery phase. This study took into account small samples and short follow-up, noting that of the 38 participants who provided the semen sample, 6 patients (15.8%) had positive results for SARS-CoV-2, including 4 of 15 patients (26, 7%) who were in the acute phase of infection and 2 out of 23 patients (8.7%) who were recovering. On the other hand, Pan et al. 2020 observed 34 Chinese adults with semen samples and reported that the virus was not detected in the analyzed material in an average of 31 days after the COVID diagnosis. In women, ACE2 expression has been reported in primordial, primary, secondary and antral follicles, in stroma and in corpus luteum, but there is no evidence for the presence of ACE2 in human oocytes. It is assumed that in the ovary there is a decrease in the expression of ACE2 after infection by SARS-CoV-2, disturbing the local RAS (renin- angiotensin-system) in the ovary, as well as disturbances in this system can lead to polycystic ovary syndrome, ovarian hyperstimulation, tumors and ectopic pregnancy. However, there is no evidence that RAS disturbances caused by SARS-CoV-2 have any adverse effect on oocyte maturation and ovarian reserve. Thus, there is a more significant amount of the ACE2 receptor in the male reproductive system compared to the female, with probably greater commitment of male fertility.

**Conclusion:** It is understood that COVID-19 affects the reproductive system of men and women, but the mechanisms by which the virus affects reproduction, and its consequences, are still uncertain. The expressive presence of the ACE2 receptor in the male reproductive system predisposes viral replication at the site, and the presence of the virus in the semen can be observed in some studies. However, the subject needs further deepening since it is not known about the number of men affected and the future impact on human reproduction. SARS-CoV-2 infect the ovary, uterus, vagina and placenta through ACE2, generating female reproductive dysfunction and possible infertility, which has not yet been proven in the literature. In this way, further studies are needed to explore the divergent pathways and cellular responses to virus infection in the male and female reproductive system. And until enough evidences have been established, cryopreservation should be encouraged.

